# The causal relationship between genetically determined telomere length and meningiomas risk

**DOI:** 10.3389/fneur.2023.1178404

**Published:** 2023-08-24

**Authors:** Weijie Yu, Yunyun Mei, Zhenwei Lu, Liwei Zhou, Fang Jia, Sifang Chen, Zhanxiang Wang

**Affiliations:** ^1^The School of Clinical Medicine, Fujian Medical University, Fuzhou, China; ^2^Department of Neurosurgery, The First Affiliated Hospital of Xiamen University, Xiamen Key Laboratory of Brain Center, Xiamen, China; ^3^Department of Neurosurgery, Fudan University Shanghai Cancer Center (Xiamen Hospital), Xiamen, China; ^4^School of Medicine, Xiamen University, Xiamen, China

**Keywords:** Mendelian randomization, meningiomas, telomere length, causality, risk

## Abstract

**Background:**

Studies have shown that longer leukocyte telomere length (LTL) is significantly associated with increased risk of meningioma. However, there is limited evidence concerning the causal association of LTL with benign and malignant meningiomas or with the location of benign tumors.

**Methods:**

We used three LTL datasets from different sources, designated by name and sample size as LTL-78592, LTL-9190, and LTL-472174. The linkage disequilibrium score (LDSC) was used to explore the association between LTL and meningioma. We utilized two-sample bidirectional Mendelian randomization (TSMR) to evaluate whether LTL is causally related to meningioma risk. We adjusted for confounders by conducting multivariable Mendelian randomization (MVMR).

**Results:**

In the LTL-78592, longer LTL was significantly associated with increased risk of malignant [odds ratio (OR) = 5.14, *p* = 1.04 × 10^−5^], benign (OR = 4.81, *p* < 0.05), benign cerebral (OR = 5.36, *p* < 0.05), and benign unspecified meningioma (OR = 8.26, *p* < 0.05). The same results were obtained for the LTL-9190. In the LTL-472174, longer LTL was significantly associated with increased risk of malignant (OR = 4.94, *p* < 0.05), benign (OR = 3.14, *p* < 0.05), and benign cerebral meningioma (OR = 3.59, *p* < 0.05). Similar results were obtained in the MVMR. In contrast, only benign cerebral meningioma displayed a possible association with longer LTL (OR = 1.01, *p* < 0.05). No heterogeneity or horizontal pleiotropy was detected.

**Conclusion:**

In brief, genetically predicted longer LTL may increase the risk of benign, malignant, and benign cerebral meningiomas, regardless of the LTL measure, in European populations.

## Introduction

Meningioma is the most common type of primary brain tumor, and most meningiomas are benign or slow-growing and easy to observe. Although mortality due to meningioma is generally low, the disease has a high incidence. The majority of meningiomas are intracranial (approximately 90%); spinal meningiomas account for approximately 10% of all meningiomas. Despite decades of research, very little is known about the etiology of meningiomas. The only currently recognized risk factor is ionizing radiation (1). Observational epidemiological studies have provided evidence that obesity (2–5), female sex hormones (6, 7), and arterial hypertension (5) are risk factors for meningioma and even associated with meningiomas in different ethnic groups (8). It has also been found that height is a potential risk factor for meningioma in European populations (2) and Israeli population (9). Hyperglycemia, type 2 diabetes mellitus (T2DM) might increase risk (10) or have an inverse effect (11, 12) on meningioma in recent studies. These observational epidemiological studies may be influenced by confounding and reverse causation, biasing findings.

The number and breadth of studies linking leukocyte telomere length (LTL) to lifelong disease risk continue to increase. LTL has been shown to be linearly inversely associated with various age-related diseases, including peripheral vascular disease, cardiovascular disease (CVD), hypertension, T2DM, and a number of neurodegenerative diseases (13–17). Another age-related disease is cancer, which is positively associated with longer LTL in cases of glioma, breast cancer, colorectal cancer, and pancreatic cancer (18–21). A recent Mendelian randomization study confirmed that genetically increased LTL is significantly associated with increased glioma risk (22). Studies have also shown that longer genotypically estimated LTL is significantly associated with increased meningioma risk (23). Other studies presented telomere length was reduced in the majority of malignant or atypical meningiomas with detectable telomerase activity (24). Telomere shortening might play an important role in the development of high grade meningioma, especially in grade III meningiomas, which have shown telomere shortening and high proliferative activity (25). Telomerase activity and its human telomerase reverse transcriptase (hTERT) mRNA expression tended to increase as the histologic grading of intracranial meningiomas increased, suggest that telomerase reactivation plays a role in the progression of meningiomas (26). These results indicate that telomere length may be a critical step in the pathogenesis of meningiomas.

To gain further insight into the causal relationship between telomere length and meningioma risk, including benign and malignant meningiomas, as well as the possible association between telomere length and tumor location, we conducted bidirectional two-sample Mendelian randomization (TSMR) and multivariable Mendelian randomization (MVMR) analyses using public genome-wide association study (GWAS) data on LTL and meningiomas.

## Materials and methods

### Ethics

Ethical approval for this study was not needed, as our analyses were based on summary statistics available in published GWASs and on data that were publicly accessible, and no individual-level data were used.

### Data sources

The first dataset was established by Codd et al. (27) through quantitative PCR assay to obtain LTL measurements in 472,174 UKB participants. 197 independent sentinel variants were identified have an association with LTL at 138 genomic loci (108 new). The GWAS ID (ieu-b-4879) of telomere length and full summary statistics are available from the MRC IEU Open GWAS Project.^1^ The second is the recent comparatively large published GWAS meta-analysis conducted by the European Network for Genetic and Genomic Epidemiology (ENGAGE), European Prospective Investigation into Cancer (EPIC)-CVD, and EPIC-Interact consortium study as one of the sources of data on LTL (28). It included 78,592 individuals (age 18–106 years) of European ancestry. Mean LTL was measured as a continuous variable by quantitative PCR and expressed as the ratio of the telomere repeat number (T) to a single-copy gene (S) (28). However, the full summary statistic was not available. To further investigate whether the method used to measure telomere length affected the results, we selected another dataset derived from a GWAS meta-analysis based on six studies in which a total of 9,190 European individuals (age 18–95 years) were enrolled as a source of data on LTL (29). In that study, telomere length was measured by the Southern blot method using the terminal restriction fragment. Age, sex, body mass index, and smoking status (pack-years) were adjusted for in this meta-analysis (29, 30). Detailed information can be found in Supplementary Table 1. The three sets of LTL datasets are designated by name and sample size as LTL-78592, LTL-472174, and LTL-9190, respectively. LTL-78592 and LTL-9190 served as replication group for the same and different LTL measurements as LTL-472174, respectively.

Genome-wide association study (GWAS) data on benign meningiomas obtained from the FinnGen database^2^ were publicly released in 2022. These data include 1,986 cases and 248,006 controls of European descent. Benign meningiomas can be divided into spinal meningiomas, cerebral meningiomas, and unspecified meningiomas based on the International Statistical Classification of Diseases and Related Health Problems, 10th Revision (ICD-10)-WHO Version for 2016. We extracted corresponding datasets from the FinnGen database for MR analysis. The first of these consisted of 185 cases and 248,637 controls, the second included 1780 cases and 248,101 controls, and the last included 315 cases and 248,611 controls. Data on malignant meningiomas were derived from the same database and included 969 cases and 238,678 controls. All controls excluded all cancers to avoid affecting the eventual results. The meningioma and LTL samples did not overlap. Detailed information can be found in Supplementary Table 1.

### Genetic instruments for telomere length

**Figure 1:**
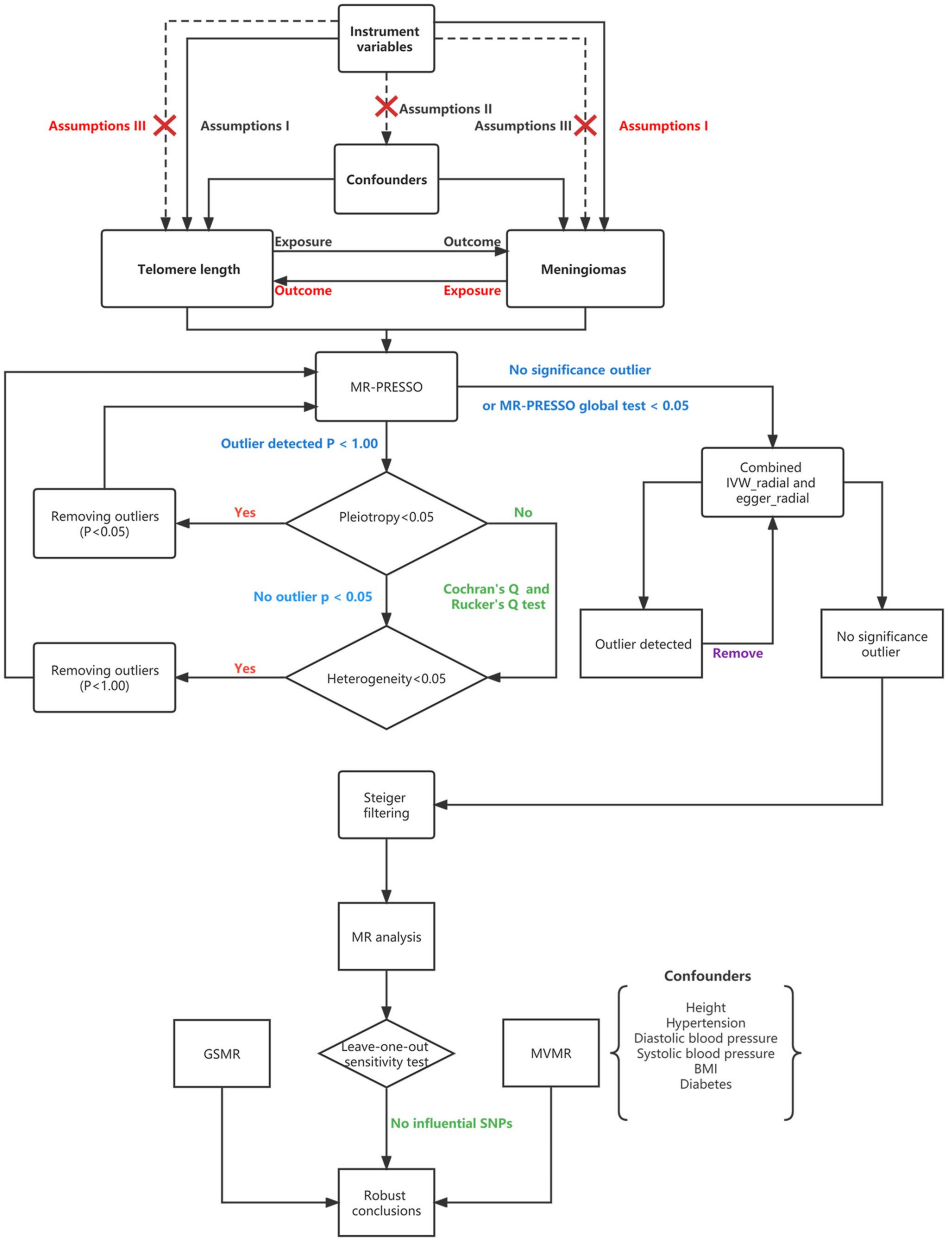
Flowchart showing Mendelian randomization. MR, Mendelian randomization; MR-PRESSO, Mendelian randomization pleiotropy residual sum and outlier; SNPs, single-nucleotide polymorphisms; BMI, body mass index; MVMR, multivariable Mendelian randomization; GSMR, generalized summary data-based Mendelian randomization; and IVW, inverse variance weighting.

Three essential model assumptions of MR analysis should be fulfilled to ensure that the instrumental variables (IVs) chosen are valid (Figure 1). Assumption I is that the selected IVs are robustly associated with the exposure; assumption II is that the IVs that may be associated with exposure are not associated with any confounders, and assumption III is that the selected IVs only affect the specific outcome exclusively through the exposure. Based on the three MR core assumptions, individual single-nucleotide polymorphisms (SNPs) were removed if the SNPs were associated with any known confounders or risk factors (e.g., height, obesity, arterial hypertension, and diabetes) and outcomes at genome-wide significance (*p* < 5 × 10^−8^) based on the PhenoScanner V2 database^3^ (31, 32). Moreover, if the SNPs were palindromic, strand-ambiguous, or associated with incompatible alleles, we removed them. Proxy SNPs were leveraged using higher linkage disequilibrium (R2 ≥ 0.9) to substitute for them through the online website SNiPA^4^ and the CEU reference population from the 1,000 Genomes Project when the SNPs were not available for the outcome. If the outcome was derived from the open GWAS database, it was proxied directly through the “extract_outcome_data” function in the TwoSampleMR package. However, when a good proxy was not available, the instrument was removed from the analysis. SNPS located in the MHC region were removed. As IVs for LTL-78592, we used 20 SNPs (see Supplementary Table 3) that were selected at the established threshold for GWAS significance (*p* < 5 × 10^−8^) and evaluated through additive models adjusted for age, sex, and cohort-specific covariates (28, 30). For the United Kingdom Biobank, we selected 154 SNPs (see Supplementary Table 3) as IVs associated with LTL-472174 using linkage disequilibrium R2 < 0.001 across a 1 Mb window, and the gene variants in LTL-472174 were adjusted for age and sex. For LTL-9190, we selected 16 SNPs (see Supplementary Table 3) as IVs to evaluate whether there was a discrepancy among the results obtained using different measurement method (33).

### Genetic instruments for the investigation of meningiomas

To make a thorough inquiry regarding the possible presence of reverse causation, we conducted reverse MR analyses in which the meningiomas were regarded as exposure and LTL was deemed the outcome. In this analysis, we used a *p* value threshold of less than 5 × 10^−6^ to select the genetic instruments because there were not enough SNPs to reach the traditional GWAS threshold. After the same steps mentioned above, only 7, 9, 6, 4, and 2 SNPs remained that fulfilled the three core assumptions, and these were selected as IVs for assessment of a possible causal relationship between the aforementioned meningiomas and LTL-472174 (see Supplementary Table 7 for details).

### Linkage disequilibrium score regression analysis

Linkage disequilibrium score (LDSC) regression analysis is a reliable and efficient method for identifying the shared genetic architecture of complex human traits; it estimates the heritability of diseases and tests their genetic correlation, primarily based on full GWAS summary data (34). In our analysis, the complete GWAS summary data of LTL and meningiomas were applied to evaluate the genetic correlations. The significance threshold was set at *p* < 0.05/5 = 0.01.

### Univariate Mendelian randomization analysis

A flowchart that describes the MR analysis is presented in Figure 1. In this analysis, the Mendelian Randomization Pleiotropy Residual Sum and Outlier (MR-PRESSO) global test first identified the outliers (SNPs with *p* values less than 0.05) and removed them if horizontal pleiotropy was present. When the number of SNPs was less than 3, MR-PRESSO did not perform well. We also tested horizontal pleiotropy using the MR–Egger regression test; in this analysis, a *p* value of the MR–Egger intercept less than 0.05 suggested horizontal pleiotropy. We conducted MR-PRESSO again after removing outliers. We then tested the between-SNP heterogeneity using inverse variance weighting (IVW) and the MR–Egger method based on the SNPs that were retained after pleiotropy correction. Cochran’s Q (for IVW) and Rucker’s Q (for MR–Egger) statistics were used to verify the presence of heterogeneity. In this step, we removed SNPs with *p* values less than 1.00 in the MR-PRESSO analysis if the heterogeneity was significant (*p* value of Cochran’s Q and Rucker’s Q statistic less than 0.05) and horizontal pleiotropy was not present (35). MR-PRESSO was then performed again. The presence of at least five SNPs is required as a prerequisite for performing the RadialMR method. If no SNPs with *p* < 1.00 were present, if none of the detected SNPs were significant in the MR-PRESSO analysis or if the global test *p* value was less than 0.05 when the MR-PRESSO analysis was performed, we then used both the radial IVW method and the Egger method to further identify outliers with *p* values less than 0.05 and removed them (36). After removing the outliers, we performed the radial IVW and Egger methods again until no outliers were identified. After the above steps, when both horizontal pleiotropy and heterogeneity were absent, we leveraged Steiger filtering; this step excluded SNPs that explained a greater proportion of the variance in the outcome than in the exposure (37). Next, we conducted the main MR analysis using the IVW method. Finally, a “leave-one-out” analysis was conducted to detect the influential SNPs; if those SNPs were absent, we regarded the conclusions as robust (38).

For exposures for which only two SNPs were available as IVs, the fixed-effects inverse-variance weighted method was used. There are also other MR methods, namely, MR–Egger, weighted median, simple mode, weighted mode, and generalized summary data-based Mendelian randomization (GSMR). The MR–Egger method allows all SNPs to be used as invalid instruments but requires variants to satisfy the InSIDE assumption; this method makes it possible to estimate appropriate causal effects in the presence of pleiotropic effects (39). The intercept in the MR–Egger regression showed evidence for pleiotropic bias and was visualized using funnel plots. When some IVs are invalid (<50%; the majority of IVs do not exhibit directional horizontal pleiotropy), the weighted median approach can be used as an alternative method to obtain a consistent estimate (40). GSMR analysis extends the MR method using all the top associated SNPs at a genome-wide significance level for exposure as IVs to test causality. Furthermore, unlike other methods, GSMR analysis accounts both for possible linkage disequilibrium between SNPs and for sampling errors in the estimated effect sizes of the instruments on exposure. However, at least 10 SNPS are required when using this method (40).

### Multivariable Mendelian randomization analysis

Given the confounders and pleiotropy, multivariable Mendelian randomization (MVMR) was performed for certain important confounders. As the main method, MVMR-IVW can insighted potential outliers and pleiotropy, and to help determine the most appropriate choice of robust method. MVMR-Lasso aims to identify and downweight outliers, performed best overall in terms of mean squared error. The MVMR-Egger estimator is robust to directional pleiotropy, even when all instruments are invalid. MVMR-PRESSO were applied to the case where pleiotropy is balanced and there are a relatively small number of outliers (41). According to a recent study, BMI, height, type 2 diabetes (T2D), and hypertension are possible risk factors for the outcome (2, 3, 5, 10). Therefore, we performed MVMR in which we adjusted for BMI, height, systolic blood pressure, diastolic blood pressure, T2D, and hypertension. The method used to screen the GWAS summary databases for confounders was the same as that used to screen the databases for exposures and outcome.

### Statistical analysis

For the MR analysis, IVs with *F* values greater than 10 were considered strong instruments that could alleviate bias from weak instruments (42). The formula used to calculate *F* values is as follows: *F* = [(N−K−1) R2]/[k(1−R2)], where R2 represents the proportion of variance explained by the genetic variants, *N* represents the sample size, and *k* represents the number of included SNPs. $R2=\overset{k}{\underset{1}{\sum }}2\beta^{2}\left(1-EAF\right)EAF,$ where EAF is the effect allele frequency and β is the estimated effect on LTL (43). Given the small number of cases in the GWAS, we calculated the statistical power for MR analysis using the mRnd website.^5^ In the interpretation of multiple testing, for forward MR analysis, *p* values below the Bonferroni-corrected threshold of 3.33 × 10^−3^ [where *p* = 0.05/15 (three exposures and five outcomes)] were treated as strong evidence of a causal association, and *p* values below 0.05 but above 3.33 × 10^−3^ were considered suggestive of an association. For reverse causality, due to multiple testing, we set the threshold to *p* less than 0.01 (0.05/5, one exposure, and five outcomes). For MVMR analysis, the conditional *F*-statistic values for all exposures were taken as the *F* statistic, the *p* value less than 0.05. All statistical analyses performed in this study were conducted using the “TwoSampleMR,” “phenoscanner,” “RadialMR,” “MendelianRandomization,” “GenomicSEM,” “GSMR,” and “MRPRESSO” packages in R software (version 4.2.2).

## Results

### Genetic correlation between LTL and meningioma-related phenotypes

As shown in Supplementary Table 2, we tested the genetic correlation between LTL and meningioma-related phenotypes in the LTL-472174 group by LDSC regression. We detected a positive genetic correlation between LTL and benign meningioma (Rg = 0.29, *p* = 1.90 × 10^−3^) and benign cerebral meningioma (Rg = 0.31, *p* = 8.19 × 10^−4^); there were irrelevant genetic correlations between LTL and malignant meningioma (Rg = 0.13, *p* = 0.105) and benign spinal meningioma (Rg = 0.01, *p* = 0.958).

### Univariate Mendelian randomization between LTL and meningioma-related phenotypes

As illustrated in the abovementioned flowchart, we filtered out the corresponding SNPs as shown in Supplementary Table 8; the specific information can be found in Supplementary Tables 4_1–4_5, 5_1–5_5, 6_1–6_5.

**Figure 2:**
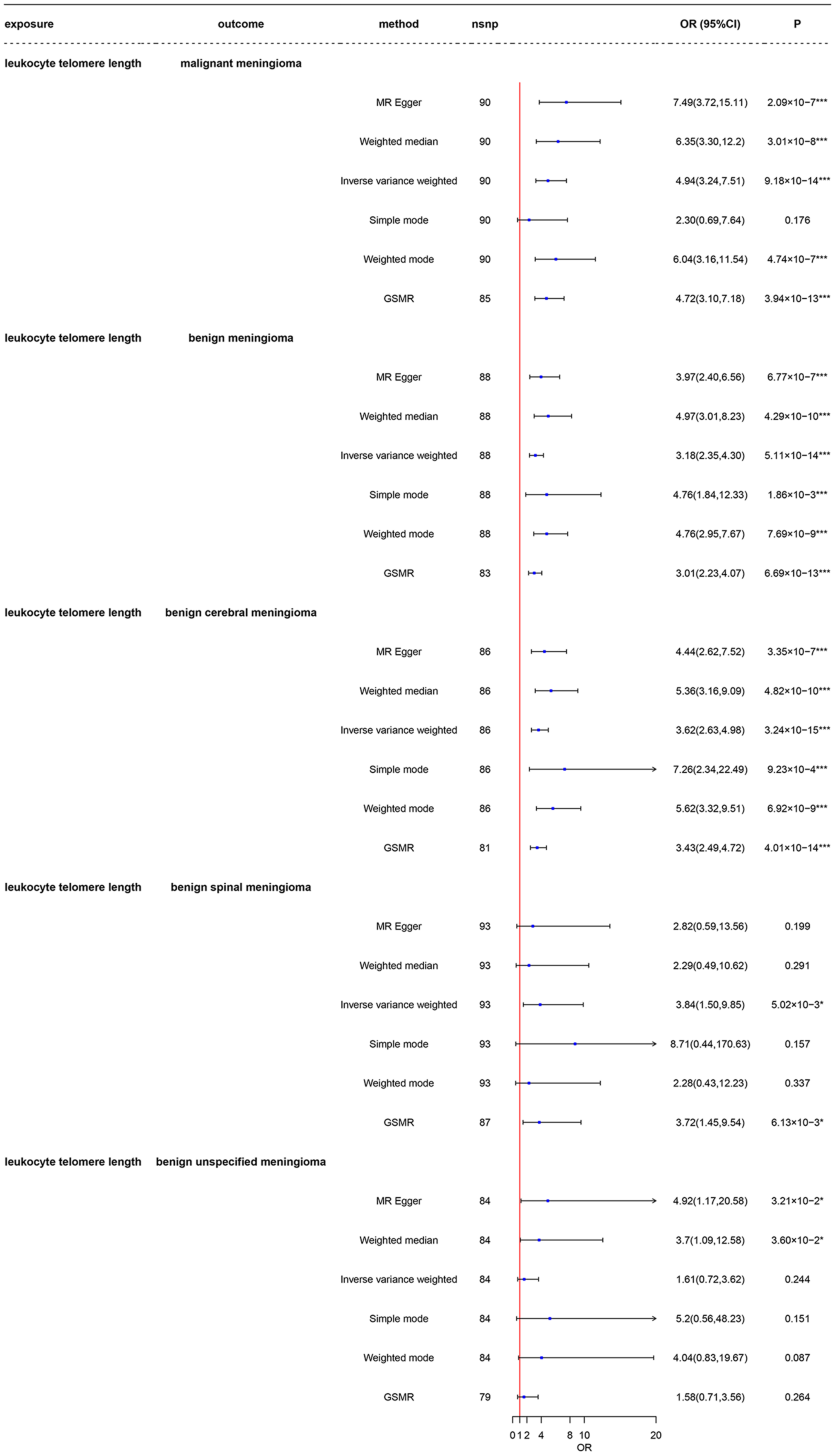
Mendelian randomization analysis of the association between leukocyte telomere length and risk of meningioma in the LTL-472147 dataset. nsnp, number of single-nucleotide polymorphisms; OR, odds ratio; CI, confidence interval; MR, Mendelian randomization; LTL, leukocyte telomere length; GSMR, generalized summary data-based Mendelian randomization. ^*^3.33 × 10^–3^ < *p* < 0.05; ^***^p < 3.33 × 10^–3^.

As shown in Figure 2 and Supplementary Table 8 in the LTL-472174, the total F-statistics for respective IVs were greater than 10 (range: 106.77–121.53) in all five groups, indicating that we effectively attenuated the bias caused by weak instrumental variables. Respective variants explained 1.89–2.40% of the variance in LTL-472174, as shown in Supplementary Table 8. Using main Mendelian randomization, we found that genetically predicted longer LTL was significantly associated with increased risk of malignant meningioma [odds ratio (OR) = 4.94, 95% CI: 3.24–7.51, *p* = 9.18 × 10^−14^], benign meningioma (OR = 3.14, CI: 2.33–4.25, *p* = 7.56 × 10^−14^), and benign cerebral meningioma (OR = 3.59, CI: 2.61–4.94, *p* = 4.25 × 10^−15^). The GSMR results are the same as the IVW results (Supplementary Figures 3_6_1–3_6_5). Longer LTL was weakly associated with the risk of benign spinal meningioma (OR = 3.84, CI: 1.50–9.85, *p* = 5.02 × 10^−3^). The four groups had more than 99.9% power to detect a causal effect between LTL and meningioma occurrence. However, longer LTL was not associated with the risk of benign unspecified meningioma (OR = 1.62, CI: 0.72–3.61, *p* = 0.243), and the power was less than 80%. The results obtained using other MR methods are shown in detail in Figure 2.

**Figure 3:**
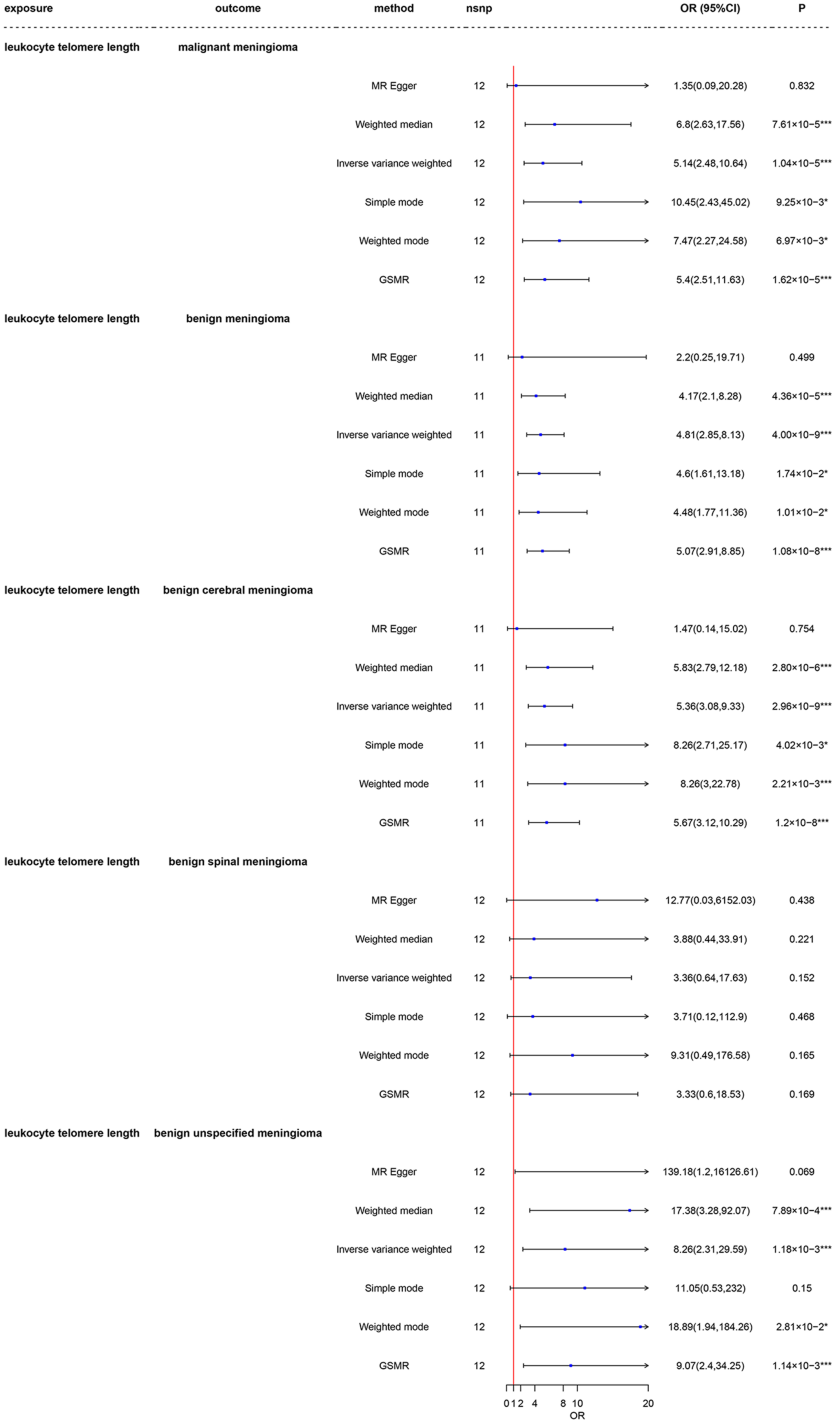
Mendelian randomization analysis of the association between leukocyte telomere length and risk of meningioma in the LTL-78592 dataset. nsnp, number of single-nucleotide polymorphisms; OR, odds ratio; CI, confidence interval; MR, Mendelian randomization; LTL, leukocyte telomere length; GSMR, generalized summary data-based Mendelian randomization. ^*^3.33 × 10^–3^ < *p* < 0.05; ^***^p < 3.33 × 10^–3^.

As shown in Figure 3 and Supplementary Table 8, the LTL-78592 serves as a replication group for the same telomere length measurement, all five groups had total F-statistics for the respective IVs that were larger than 10 (range: 52.77–54.70), indicating that the instruments were strongly associated with the exposure and with less bias. Respective variants explained 0.76–0.80% of the variance in LTL-78592, as shown in Supplementary Table 8. Using main Mendelian randomization with the IVW method, we found that a genetically predicted longer LTL was significantly associated with increased risk of malignant meningioma (OR = 5.14, CI: 2.48–10.64, *p* = 1.04 × 10^−5^), benign meningioma (OR = 4.81, CI: 2.85–8.12, *p* = 4.00 × 10^−9^), benign cerebral meningioma (OR = 5.36, CI: 3.08–9.33, *p* = 2.69 × 10^−9^), and benign unspecified meningioma (OR = 8.26, CI: 2.31–29.59, *p* = 1.18 × 10^−3^). The results obtained using GSMR are similar to those obtained using IVW (Supplementary Figures 1_6_1–1_6_5). Longer LTL was not associated with risk of benign spinal meningioma (OR = 3.36, CI: 0.63–17.63, *p* = 0.152). All five groups had more than 80% power to detect a causal effect between LTL and meningioma occurrence at a significance level of 0.05. MR–Egger provided less precise estimates, but those estimates were in a direction consistent with the estimates obtained using the other four methods. The results obtained using the other MR methods are shown in detail in Figure 3.

**Figure 4:**
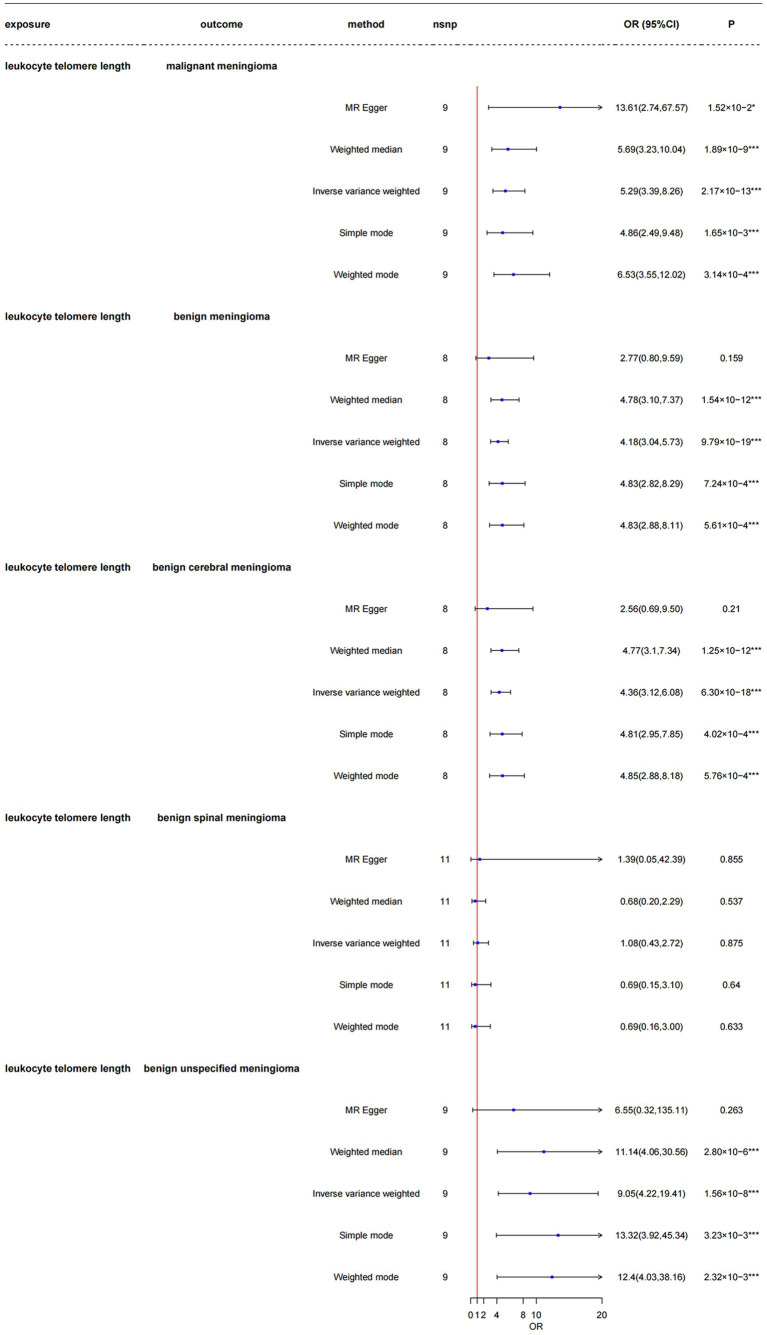
Mendelian randomization analysis of the association between leukocyte telomere length and risk of meningioma in the LTL-9190 dataset. nsnp, number of single-nucleotide polymorphisms; OR, odds ratio; CI, confidence interval; MR, Mendelian randomization; LTL, leukocyte telomere length. ^*^3.33 × 10^–3^ < *p* < 0.05; ^***^p < 3.33 × 10^–3^.

As shown in Figure 4 and Supplementary Table 8, the LTL-9190 serves as a replicate group for different telomere length measurements, the total F-statistics for respective IVs were greater than 10 (range: 21.59–23.41) in all five groups, indicating that the instruments were strongly associated with the exposure and with less bias. Respective variants explained 2.00–2.49% of the variance in LTL-9190, as shown in Supplementary Table 8. In the main MR analysis, which was conducted using the inverse-variance weighted method, we found that genetically predicted longer LTL was significantly associated with increased risk of malignant meningioma [odds ratio (OR) = 5.29, 95% CI: 3.39–8.26, *p* = 2.17 × 10^−13^], benign meningioma (OR = 4.18, CI: 3.04–5.73, *p* = 9.79 × 10^−19^), benign cerebral meningioma (OR = 4.36, CI: 3.12–6.08, *p* = 6.30 × 10^−18^), and benign unspecified meningioma (OR = 9.05, CI: 4.22–19.41, *p* = 1.56 × 10^−8^). The four groups had 100% power to detect a causal effect between LTL and meningioma occurrence. However, longer LTL was not associated with risk of benign spinal meningioma (OR = 1.08, CI: 0.43–2.72, *p* = 0.875), and the power in this group was less than 80%. The results obtained using other MR methods are shown in detail in Figure 4.

In the above process, no heterogeneity or horizontal pleiotropy was detected. MR-PRESSO had a *p* value greater than 0.05, and the leave-one-out result showed that there was no single SNP associated with the correlation between LTL and risk of meningioma in any of the three LTL datasets (Supplementary Table 8). We utilized scatter and forest plots to visualize the relationship between each genetic variant and the occurrence of meningioma (Supplementary Figures 1_1–1_5, 2_1–2_5, 3_1–3_5). No evidence of horizontal pleiotropy was noted in the MR–Egger regression intercept analysis (Supplementary Table 8), the results of which were visualized using a funnel plot (Supplementary Figures 1_1–1_5, 2_1–2_5, 3_1–3_5). The results of the leave-one-out analysis indicated that there was no single genetic variant that altered the causality (Supplementary Figures 1_1–1_5, 2_1–2_5, 3_1–3_5) in any of the three datasets.

Evaluation of the reverse causality relationship between LTL and meningioma occurrence showed that only benign cerebral meningioma had a possible association with longer LTL (OR = 1.01, CI: 1.00–1.02, *p* = 3.61 × 10^−2^); the other four types of meningioma did not, as shown in Supplementary Table 11. Since only two SNPs were included in the benign unspecified meningioma group, it was not possible to assess horizontal pleiotropy and perform the leave-one-out method in that group. No heterogeneity or horizontal pleiotropy was detected in the other groups. MR-PRESSO yielded a *p* value greater than 0.05, and pleiotropy was not detected (Supplementary Table 9). We utilized scatter and forest plots to visualize the relationship between each genetic variant and meningioma occurrence (Supplementary Figures 4_1–4_5). The results were visualized in a funnel plot (Supplementary Figures 4_1–4_5). Based on the results of the leave-one-out analysis, there was no single genetic variant that altered the causality (Supplementary Figures 4_1–4_4). The other sensitivity analysis results are presented in Supplementary Table 9.

### Multivariable Mendelian randomization between LTL and meningioma-related phenotypes

The characteristics of the SNPs of the included multivariate Mendelian randomization are listed in Supplementary Tables 10_1–10_5. After adjustment for BMI, height, systolic blood pressure, diastolic blood pressure, T2D, and hypertension (Table 1), LTL remained causally related to the occurrence of malignant meningioma (OR = 3.61, CI: 2.14–6.09, *p* = 1.78 × 10^−6^), benign meningioma (OR = 2.45, CI: 1.66–3.62, *p* = 3.77 × 10^−7^), and benign cerebral meningioma (OR = 3.12, CI: 2.13–4.59, *p* = 8.28 × 10^−9^); however, there was no causal relationship with the occurrence of benign spinal meningioma (OR = 1.42, CI: 0.44–4.60, *p* = 0.563) or benign unspecified meningioma (OR = 1.57, CI: 0.64–3.90, *p* = 0.327).

**Table 1:** Results of multivariable Mendelian randomization analysis between leukocyte telomere length and meningioma after adjustment for BMI, height, systolic blood pressure, diastolic blood pressure, T2D, and hypertension.

Outcome	Exposure	tnSNPs	OR (95%CI)	*p* value	Conditional F-statistics	Q	Q_pval	Multivariable egger intercept		
								Beta	SE	*p* value
Malignant meningioma		966				956.940	0.513	1.39E-03	2.47E-03	0.573
	BMI		1.54 (0.99–2.38)	0.055	13.249					
	Height		1.23 (0.98–1.55)	0.082	19.357					
	Systolic blood pressure		1.00 (0.96–1.05)	0.934	11.541					
	Diastolic blood pressure		1.03 (0.96–1.11)	0.451	12.652					
	Telomere length		3.61 (2.14–6.09)	1.78E-06	17.619					
	T2D		0.87 (0.75–1.01)	0.063	18.843					
	Hypertension		0.04 (0.001–1.49)	0.082	10.443					
Benign meningioma		954				921.567	0.709	1.33E-03	1.75E-03	0.447
	BMI		1.24 (0.91–1.69)	0.165	11.964					
	Height		1.34 (1.14–1.58)	3.95E-04	19.478					
	Systolic blood pressure		1.00 (0.97–1.03)	0.966	12.212					
	Diastolic blood pressure		0.99 (0.94–1.04)	0.548	15.758					
	Telomere length		2.45 (1.66–3.62)	3.77E-07	12.271					
	T2D		0.95 (0.86–1.05)	0.301	14.753					
	Hypertension		0.28 (0.02–3.32)	0.311	10.587					
Benign cerebral meningioma		953				911.533	0.777	8.11E-04	1.85E-03	0.662
	BMI		1.23 (0.89–1.70)	0.209	29.85					
	Height		1.35 (1.14–1.61)	5.42E-04	40.479					
	Systolic blood pressure		1.00 (0.96–1.03)	0.814	26.371					
	Diastolic blood pressure		0.99 (0.94–1.05)	0.759	34.98					
	Telomere length		3.12 (2.13–4.59)	8.28E-09	22.139					
	T2D		0.93 (0.84–1.04)	0.202	28.18					
	Hypertension		0.44 (0.03–6.00)	0.536	17.976					
Benign spinal meningioma		970				942.600	0.666	-4.43E-03	5.64E-03	0.433
	BMI		0.85 (0.32–2.28)	0.742	20.67					
	Height		1.54 (0.91–2.61)	0.106	48.416					
	Systolic blood pressure		0.99 (0.89–1.09)	0.796	41.974					
	Diastolic blood pressure		1.00 (0.85–1.18)	0.972	45.842					
	Telomere length		1.42 (0.44–4.60)	0.563	9.481					
	T2D		0.91 (0.65–1.27)	0.576	22.733					
	Hypertension		0.07 (0–218.67)	0.522	7.858					
Benign unspecified meningioma		970				949.927	0.603	6.68E-03	4.33E-03	0.123
	BMI		1.93 (0.90–4.13)	0.091	21.372					
	Height		1.10 (0.74–1.65)	0.634	48.276					
	Systolic blood pressure		1.01 (0.94–1.09)	0.760	43.258					
	Diastolic blood pressure		0.97 (0.86–1.10)	0.640	45.843					
	Telomere length		1.57 (0.64–3.90)	0.327	9.417					
	T2D		1.00 (0.78–1.30)	0.988	20.054					
	Hypertension		0.51 (0.001–237.25)	0.828	8.747					

BMI, body mass index; T2D, type 2 diabetes; tnSNPs, the total number of single-nucleotide polymorphisms.

## Discussion

In the present study, we used MR to explore the causal relationship between telomere length and the occurrence of meningioma and evaluated differences in benignity and malignancy, tumor location, and in the results obtained using different methods to measure telomere length. We also assessed the inverse causality association between telomere length and meningioma risk. In the LTL-472147 group, we investigated the genetic correlation and causal association between LTL and meningioma occurrence. The findings obtained through LDSC regression indicate a significantly genetic correlation between longer LTL and risk of benign meningioma and benign cerebral meningioma. We also found that longer LTL may be weakly associated with the occurrence of benign spinal meningioma but no evidence for an association with benign unspecified meningioma. The reason for this result, which contrasts with the results obtained using the other two datasets, may be the low number of cases of benign spinal meningioma and benign unspecified meningioma in this group. Further study using updated data obtained in large genetic studies is warranted to confirm the results of our MR study. The results obtained from datasets measuring telomere length whether using the same or different methods showed a consistent causal relationship between LTL and malignant meningioma, benign meningioma and benign cerebral meningioma occurrence, suggesting that the method used to measure telomere length may have no significant effect on detection of the forward causal relationship between telomere length and meningioma risk. Although the proportion of phenotypic variance explained by LTL-9190 was higher than that explained by LTL-78592, both LTL-9190 and LTL-78592 had power of greater than 80% for malignant, benign, benign cerebral, and benign unspecified meningiomas, suggesting that the results still have a high degree of confidence. Regardless, longer LTL significantly increased the risk of benign cerebral meningioma, benign meningioma, and malignant meningioma in all three datasets analyzed. To increase the robustness of our results, we adjusted for six confounders using multivariate Mendelian randomization in the LTL-472174, and the results obtained after adjustment led us to the same conclusion. This overall result may be related to the following mechanism. As cells replicate their DNA during mitosis, telomeres are shortened due to the inherent limitations of the DNA replication process. It termed the “end replication problem” (44). Longer telomeres allow more cells divides before reaching replicative senescence, thus potentially leading to mutations that allow cells to grow indefinitely and undergo malignant transformation (33). How to maintenance of telomere length is essential for cancer cells to overcome cellular senescence induced by telomere shortening. Telomeres are also regulated by Telomerase reverse transcriptase (TERT). TERT is the rate-limiting catalytic subunit of telomerase, an RNA-dependent DNA polymerase that lengthens telomeric DNA to maintain telomere homeostasis. TERT expression in most cells will silently express and enter a senescent state as telomeres shorten because it is otherwise very tightly controlled in normal cells. While in highly replicative cells, the TERT expression maintains telomere length. Such mutations have been found in meningioma, glioblastoma, medulloblastoma, and non-central nervous system (CNS) cancers (45–48). However, Keiko et al. (25) have reported that telomere shortening and high proliferation activity are pivotal for the development of high-grade meningiomas, while just a small number of grades I and II meningiomas with telomere shortening but lacked high proliferation activity and atypical mitosis. Atypical mitosis might present telomere deficiency or mutations in genes involved in cell proliferation. Similar results were shown in other study, the length of telomere from primary meningioma was shorter compared to normal cells (49). Although the sample cohort is not very large, we cannot ignore the possible shortening of telomere length in high-grade meningiomas. Meanwhile, early telomere analysis can potentially identify high-risk patients.

In contrast to the inevitable bias that occurs in observational studies, our two-sample MR study has a large sample size and sufficient statistical power to allow us to address the issue of unmeasured or uncontrolled confounders through reanalysis of the GWAS data. Although the exact role of LTL in meningioma occurrence has not been clearly defined, our findings suggest that LTL is a common feature of these diseases. Longer telomeres are a key feature of tumor cells and are not typically observed in normal cells (50). Therefore, therapeutic strategies that target telomere elongation would theoretically act specifically on tumor cells and have minimal toxic side effects on normal cells. However, few drugs that affect telomere elongation have been studied.

Our study has some limitations. First, meningiomas, the most common type of primary intracranial tumor, are more common in females than in males (51), and we were not able to investigate the sex-specific effects of LTL on meningiomas because of the lack of availability of corresponding GWASs. Likewise, we were unable to use other meningioma datasets to further validate the analysis due to the lack of other complete meningioma GWAS datasets. Second, while we attempted to eliminate the effects of pleiotropic and heterogeneous factors and confounders, some effects of these factors or confounders may remain. Third, although lowering the threshold of the *p* value in the reverse MR increases the number of IVs, it makes the validation of IV assumptions challenging and may consequently lead to biased causal estimates or false-positive causal relationships. Fourth, because all participants in the GWASs were of European ancestry, the conclusions may not apply to other races. Fifth, telomere length was measured in leukocytes, not meninges, and variations in telomere length explained only a small proportion of the variance in each of the three LTL datasets. In addition to LTL-472174, the full summary statistic of the other two datasets was not available, so that we cannot rule out that there is no correlation between these three datasets. The similarity of MR results may be influenced in part by correlations between the three LTL datasets. Sixth, exploring the association between genetically predicted LTL and the occurrence of specific types of meningioma remains a challenge in the current MR analysis due to the lack of availability of GWAS datasets that include molecular or histological typing of meningiomas. Therefore, in future GWASs, subgroup analysis based on meningioma typing is recommended.

In conclusion, there is a certain causal relationship between LTL and meningioma. Presently, therapies targeting for adjustment of LTL will have an impact on the prognosis of meningioma. As our understanding of the role of telomere length in meningioma biology improves, it could pave the way for better finding potential prognostic biomarkers, and more therapeutic opportunities will likely be identified.

## Data availability statement

The original contributions presented in the study are included in the article/Supplementary material, further inquiries can be directed to the corresponding authors.

## Author contributions

WY, YM, and ZW: study design. WY, YM, ZL, LZ, and FJ: acquisition of datasets. WY and YM: data analysis and manuscript drafting. SC and ZW: manuscript revision. WY, YM, ZL, LZ, FJ, SC, and ZW: final approval of manuscript. All authors contributed to the article and approved the submitted version.

## Funding

This study was funded by the Natural Science Foundation of China (82072777).

## Conflict of interest

The authors declare that the research was conducted in the absence of any commercial or financial relationships that could be construed as a potential conflict of interest.

## Publisher’s note

All claims expressed in this article are solely those of the authors and do not necessarily represent those of their affiliated organizations, or those of the publisher, the editors and the reviewers. Any product that may be evaluated in this article, or claim that may be made by its manufacturer, is not guaranteed or endorsed by the publisher.

## Footnotes

^1^https://gwas.mrcieu.ac.uk/

^2^https://r7.finngen.fi/

^3^http://www.phenoscanner.medschl.cam.ac.uk/

^4^http://snipa.helmholtz-muenchen.de/snipa3/

^5^https://shiny.cnsgenomics.com/mRnd/
