# Supplementary material for: The causal relationship between genetically determined telomere length and meningiomas risk
Source: Front Neurol. 2023 Aug 24;14:1178404. doi: 10.3389/fneur.2023.1178404 (PMC10484632; doi:10.3389/fneur.2023.1178404)
Supplement: Supplementary file 2 [file Data_Sheet_1.pdf]

Supplementary Material

The causal relationship between genetically determined telomere length and meningiomas risk

Supplementary Figures

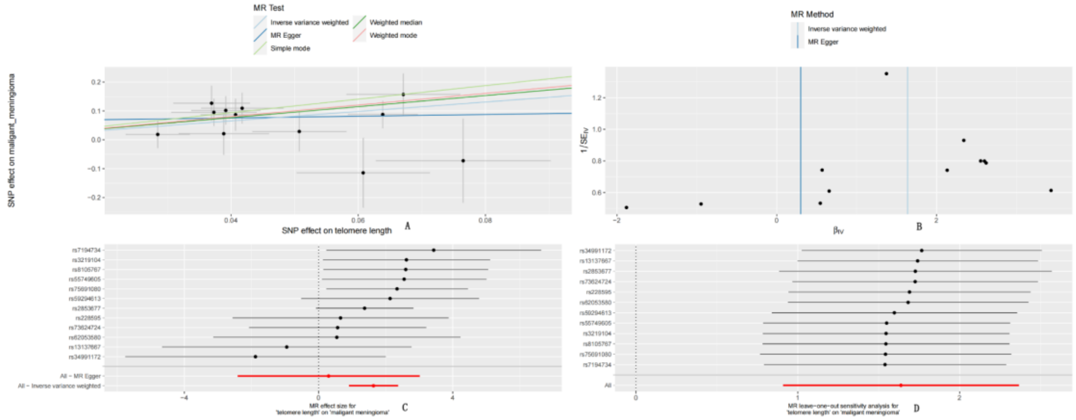

Supplementary figure 1_1 Illustrations of forward Mendelian randomization and sensitivity analysis between LTL-78592 and malignant meningiomas. A: scatter plot; B: funnel plot; C: forest plot; D: leave-one-out analysis.

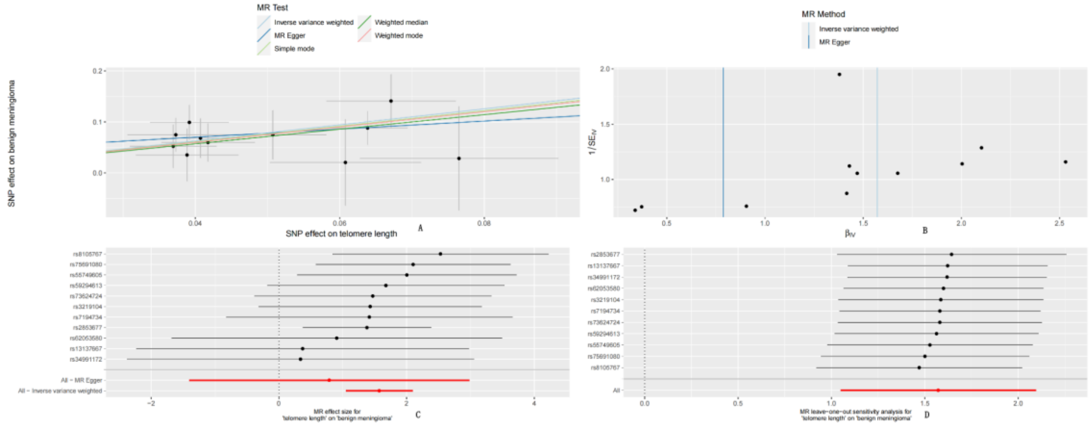

Supplementary figure 1_2 Illustrations of forward Mendelian randomization and sensitivity analysis between LTL-78592 and benign meningiomas. A: scatter plot; B: funnel plot; C: forest plot; D: leave-one-out analysis.

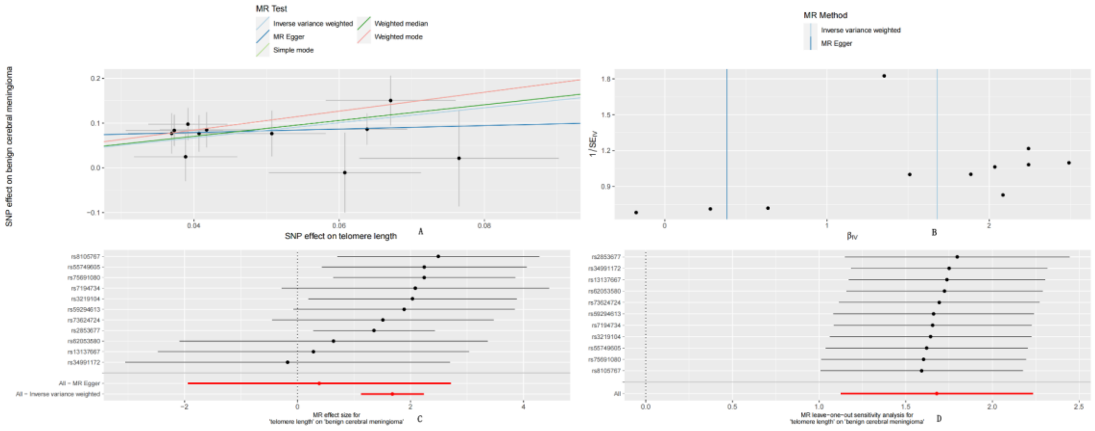

Supplementary figure 1_3 Illustrations of forward Mendelian randomization and sensitivity analysis between LTL-78592 and benign cerebral meningiomas. A: scatter plot; B: funnel plot; C: forest plot; D: leave-one-out analysis.

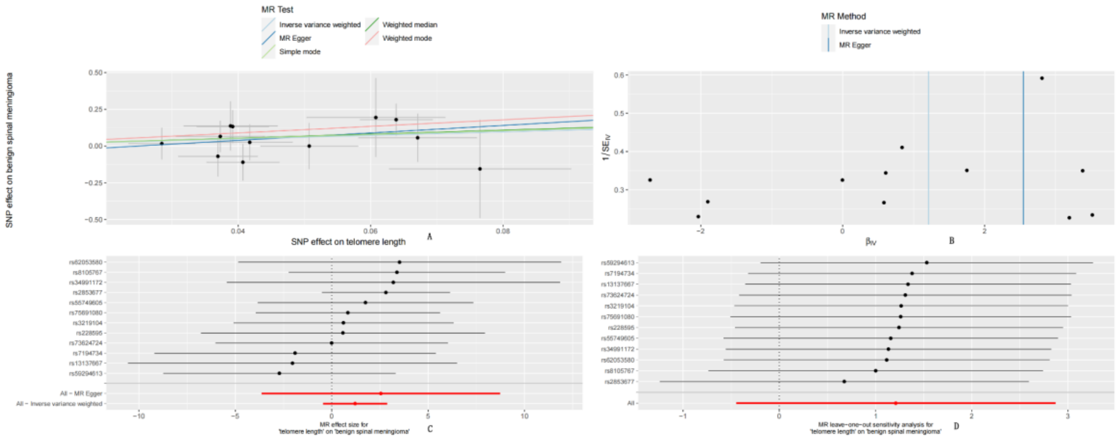

Supplementary figure 1_4 Illustrations of forward Mendelian randomization and sensitivity analysis between LTL-78592 and benign spinal meningiomas. A: scatter plot; B: funnel plot; C: forest plot; D: leave-one-out analysis.

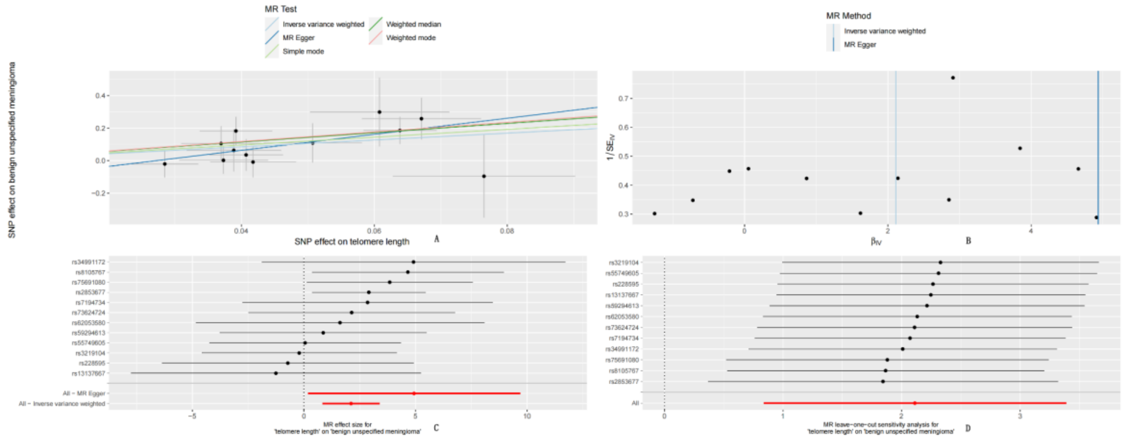

Supplementary figure 1_5 Illustrations of forward Mendelian randomization and sensitivity analysis between LTL-78592 and benign unspecified meningiomas. A: scatter plot; B: funnel plot; C: forest plot; D: leave-one-out analysis.

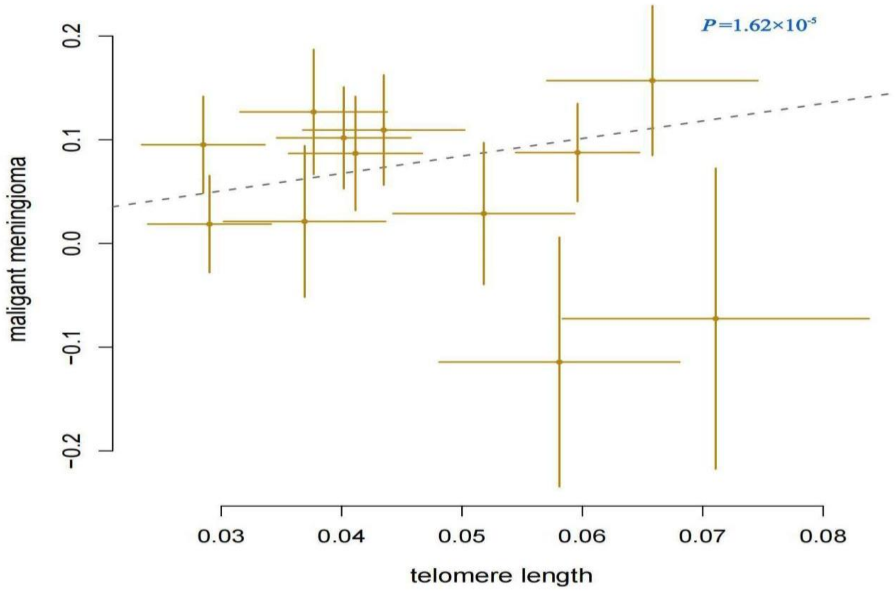

Supplementary figure 1_6_1 Illustration of forward Generalized summary data-based Mendelian randomization between LTL-78592 and malignant meningiomas

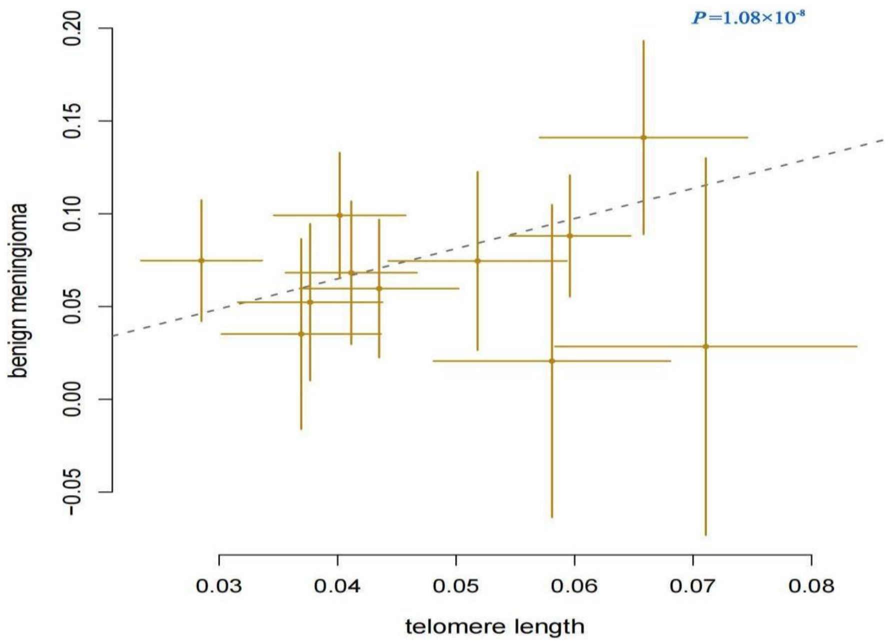

Supplementary figure 1_6_2 Illustration of forward Generalized summary data-based Mendelian randomization between LTL-78592 and benign meningiomas

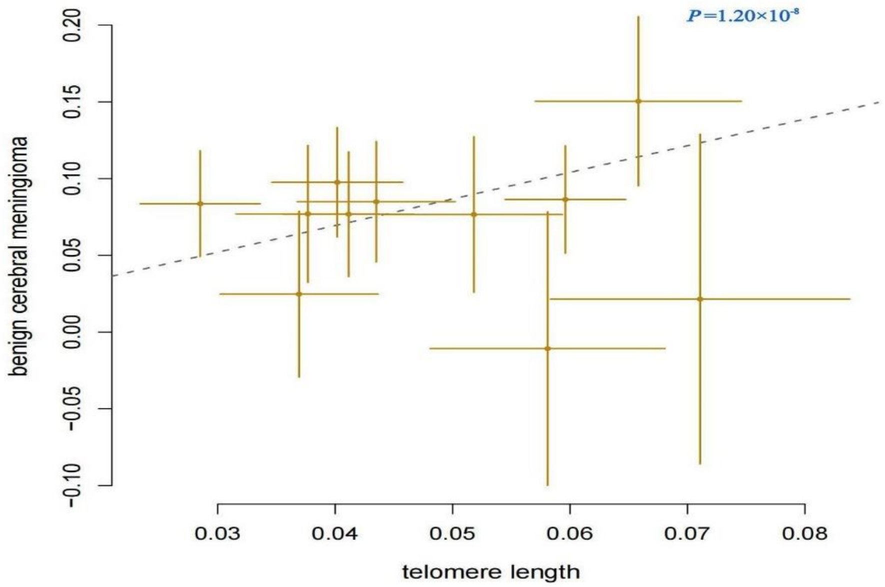

Supplementary figure 1_6_3 Illustration of forward Generalized summary data-based Mendelian randomization between LTL-78592 and benign cerebral meningiomas

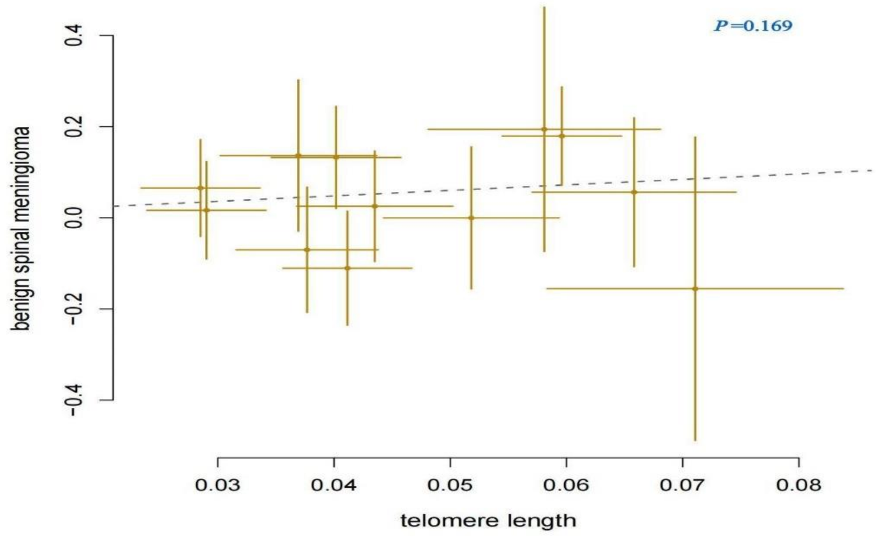

Supplementary figure 1_6_4 Illustration of forward Generalized summary data-based Mendelian randomization between LTL-78592 and benign spinal meningiomas

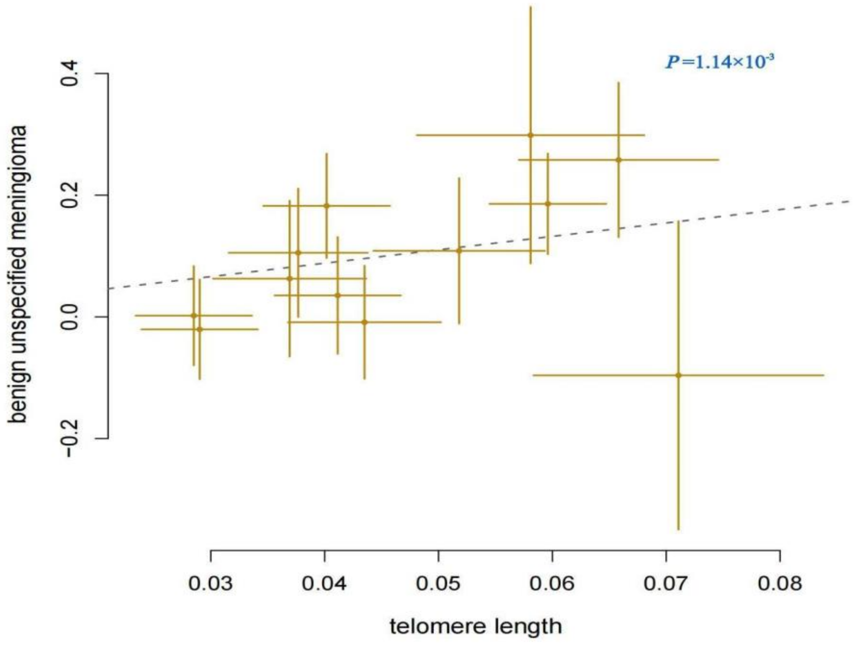

Supplementary figure 1_6_5 Illustration of forward Generalized summary data-based Mendelian randomization between LTL-78592 and benign unspecified meningiomas

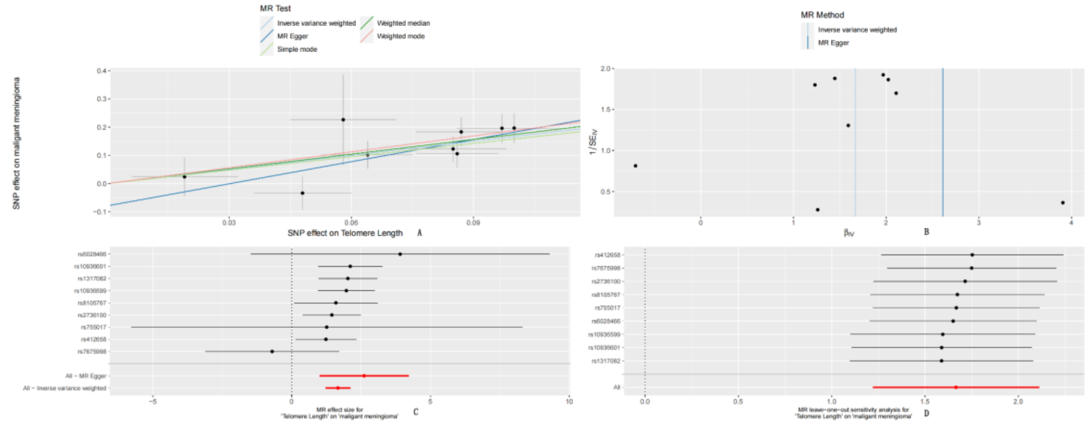

Supplementary figure 2_1 Illustrations of forward Mendelian randomization and sensitivity analysis between LTL-9190 and malignant meningiomas. A: scatter plot; B: funnel plot; C: forest plot; D: leave-one-out analysis.

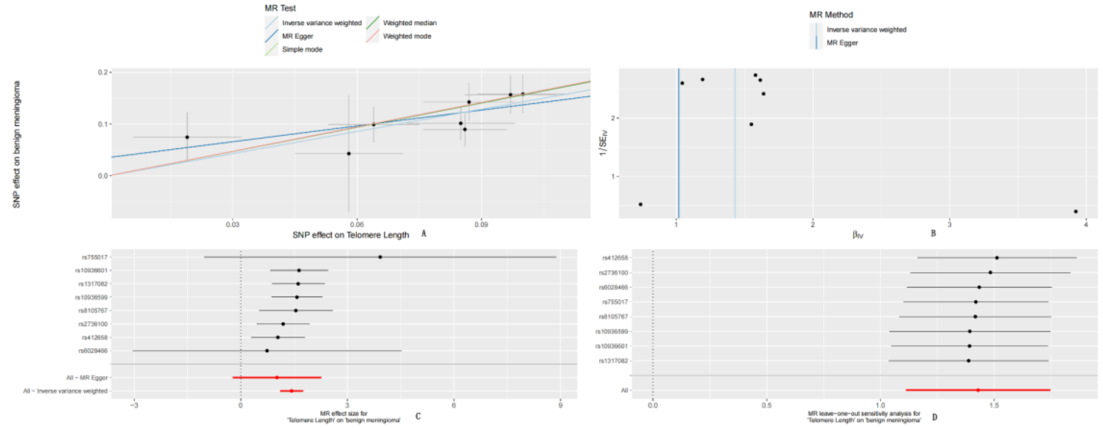

Supplementary figure 2_2 Illustrations of forward Mendelian randomization and sensitivity analysis between LTL-9190 and benign meningiomas. A: scatter plot; B: funnel plot; C: forest plot; D: leave-one-out analysis.

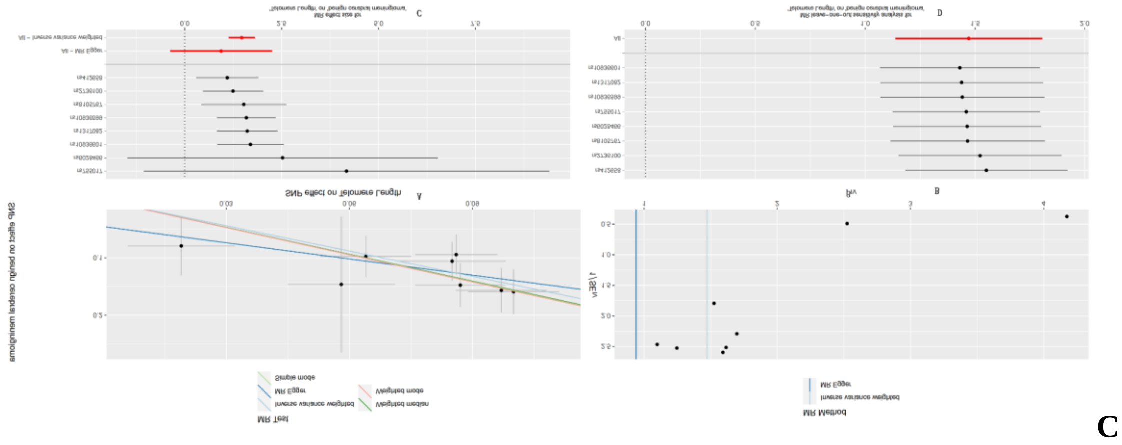

Supplementary figure 2_3 Illustrations of forward Mendelian randomization and sensitivity analysis between LTL-9190 and benign cerebral meningiomas. A: scatter plot; B: funnel plot; C: forest plot; D: leave-one-out analysis.

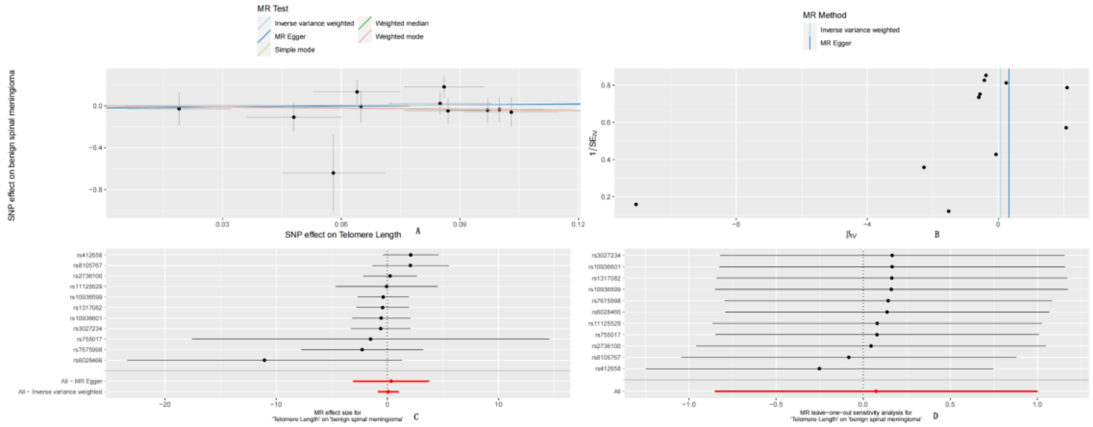

Supplementary figure 2_4 Illustrations of forward Mendelian randomization and sensitivity analysis between LTL-9190 and benign spinal meningiomas. A: scatter plot; B: funnel plot; C: forest plot; D: leave-one-out analysis.

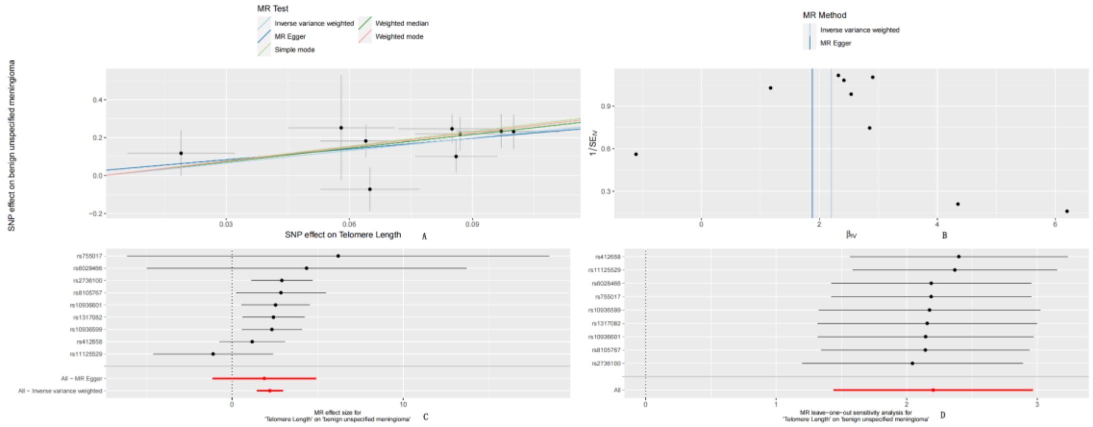

Supplementary figure 2_5 Illustrations of forward Mendelian randomization and sensitivity analysis between LTL-9190 and benign spinal meningiomas. A: scatter plot; B: funnel plot; C: forest plot; D: leave-one-out analysis.

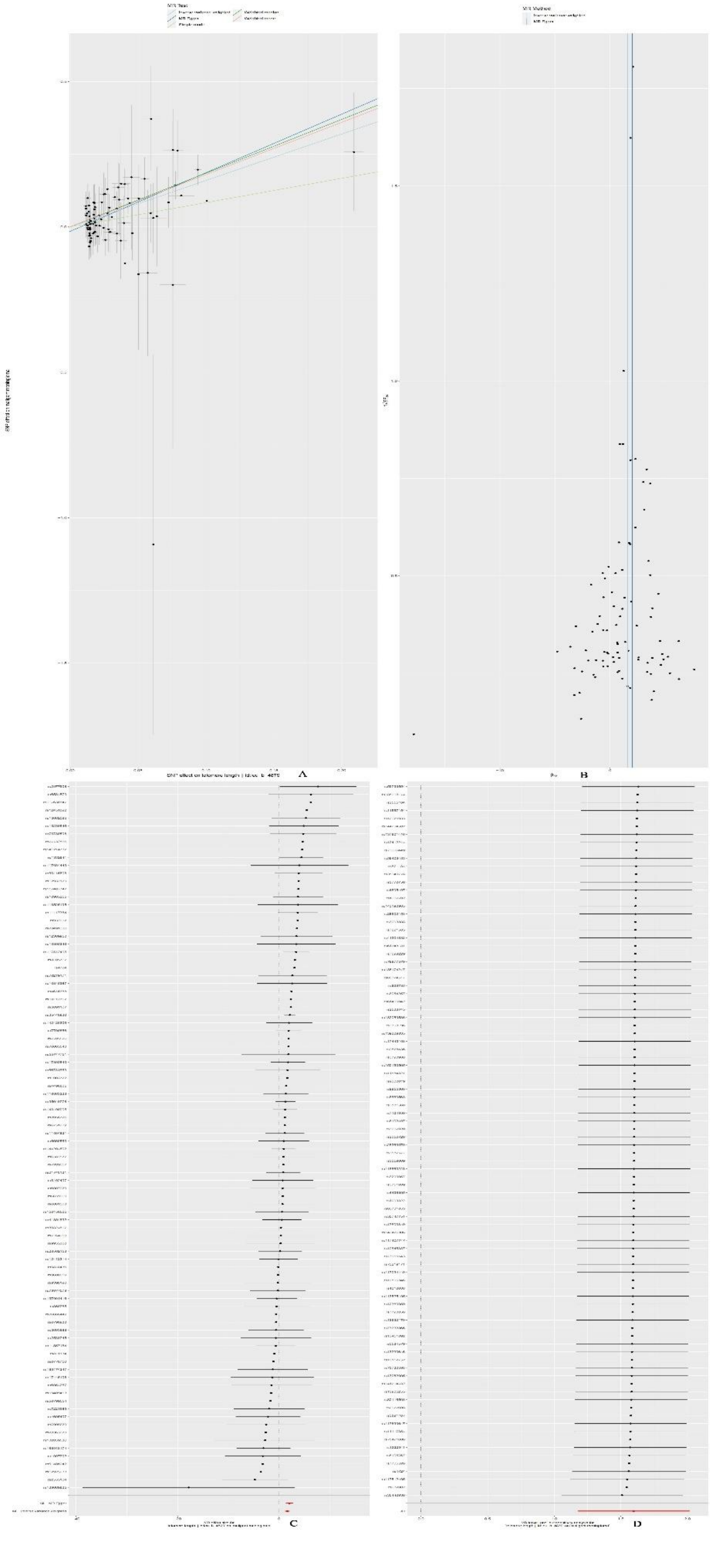

Supplementary figure 3_1 Illustrations of forward Mendelian randomization and sensitivity analysis between LTL-472174 and malignant meningiomas. A: scatter plot; B: funnel plot; C: forest plot; D: leave-one-out analysis.

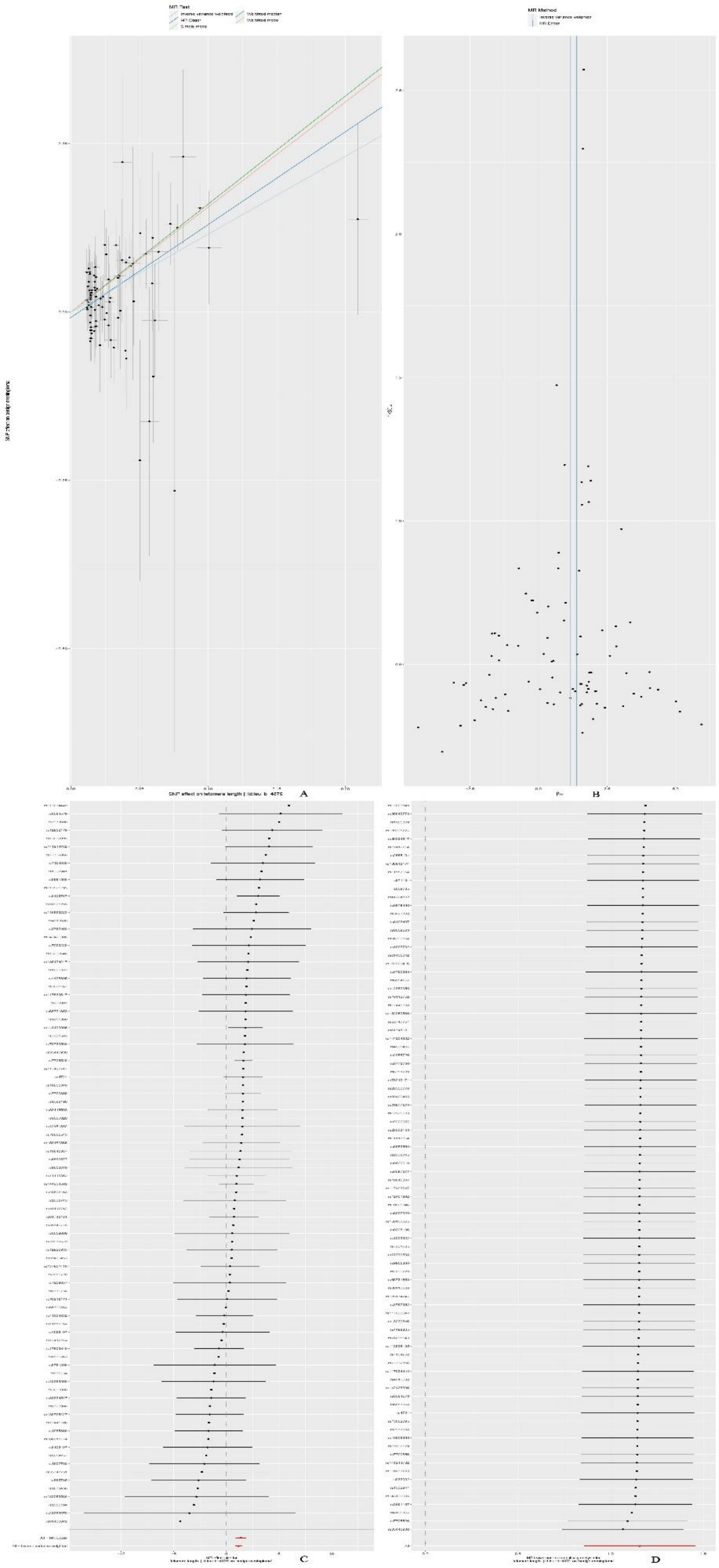
A
B
C
D

Supplementary figure 3_2 Illustrations of forward Mendelian randomization and sensitivity analysis between LTL-472174 and benign meningiomas. A: scatter plot; B: funnel plot; C: forest plot; D: leave-one-out analysis.

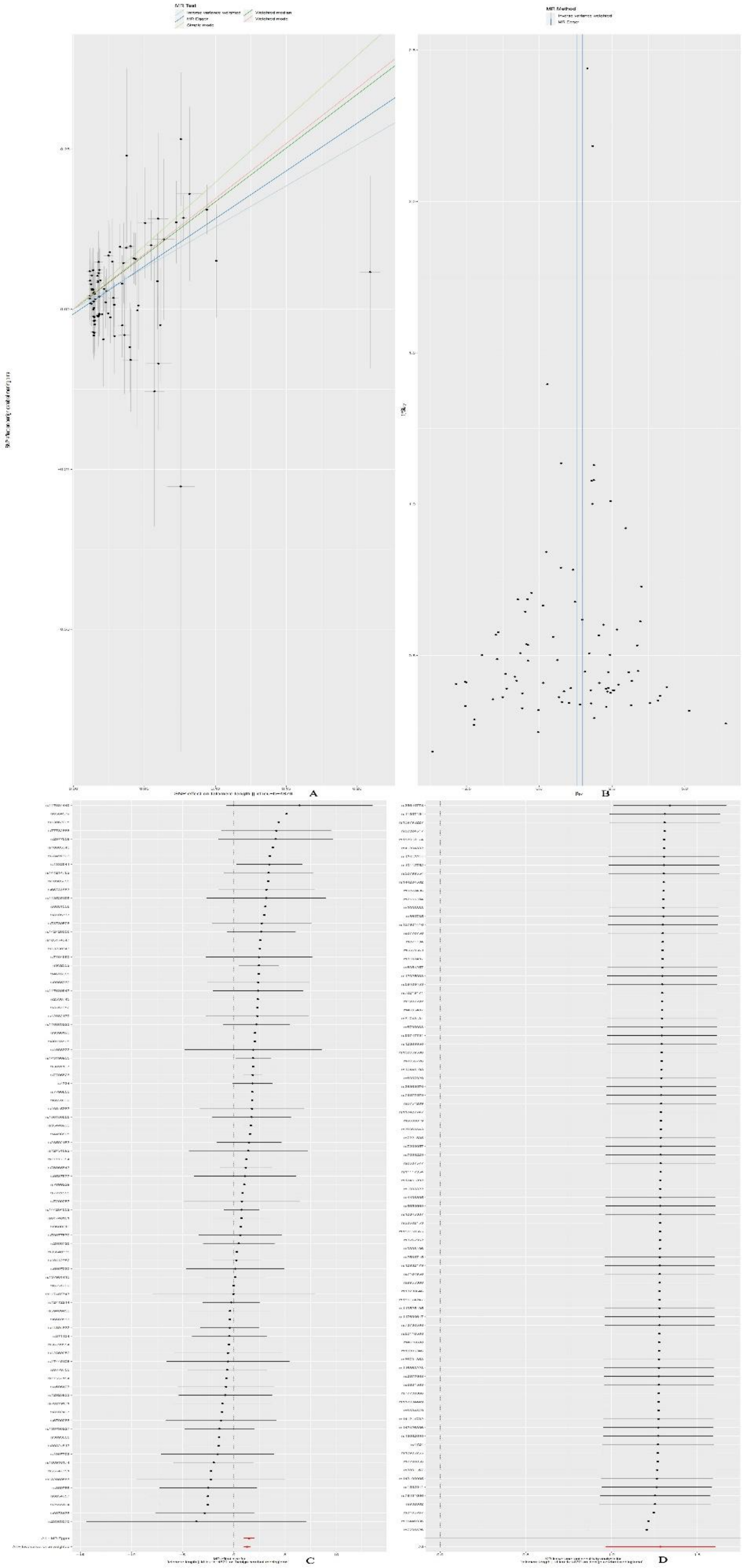
A
B
C
D

Supplementary figure 3_3 Illustrations of forward Mendelian randomization and sensitivity analysis between LTL-472174 and benign cerebral meningiomas. A: scatter plot; B: funnel plot; C: forest plot; D: leave-one-out analysis.

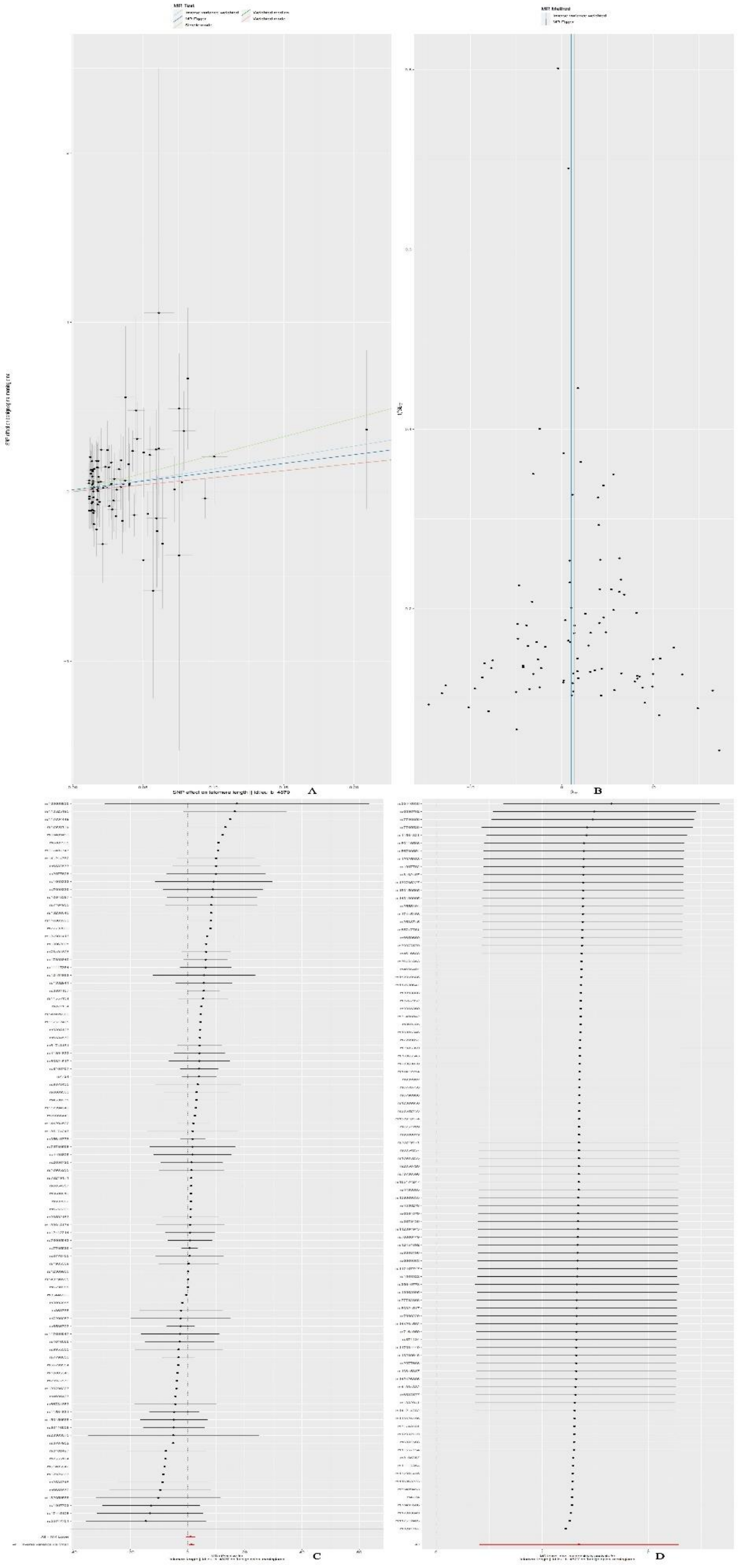
A
B
C
D

Supplementary figure 3_4 Illustrations of forward Mendelian randomization and sensitivity analysis between LTL-472174 and benign spinal meningiomas. A: scatter plot; B: funnel plot; C: forest plot; D: leave-one-out analysis.

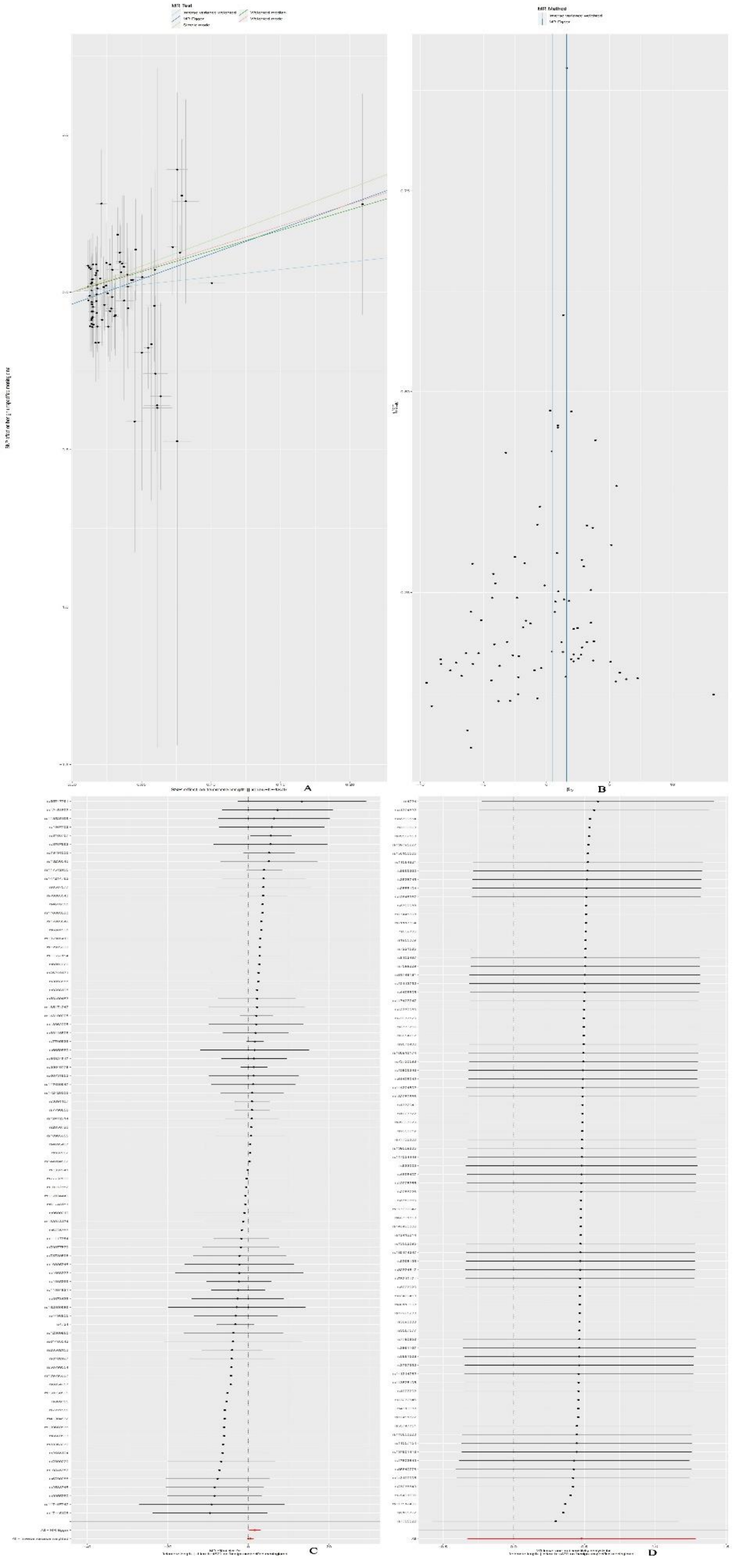

A B C D

Supplementary figure 3_5 Illustrations of forward Mendelian randomization and sensitivity analysis between LTL-472174 and benign unspecified meningiomas. A: scatter plot; B: funnel plot; C: forest plot; D: leave-one-out analysis.

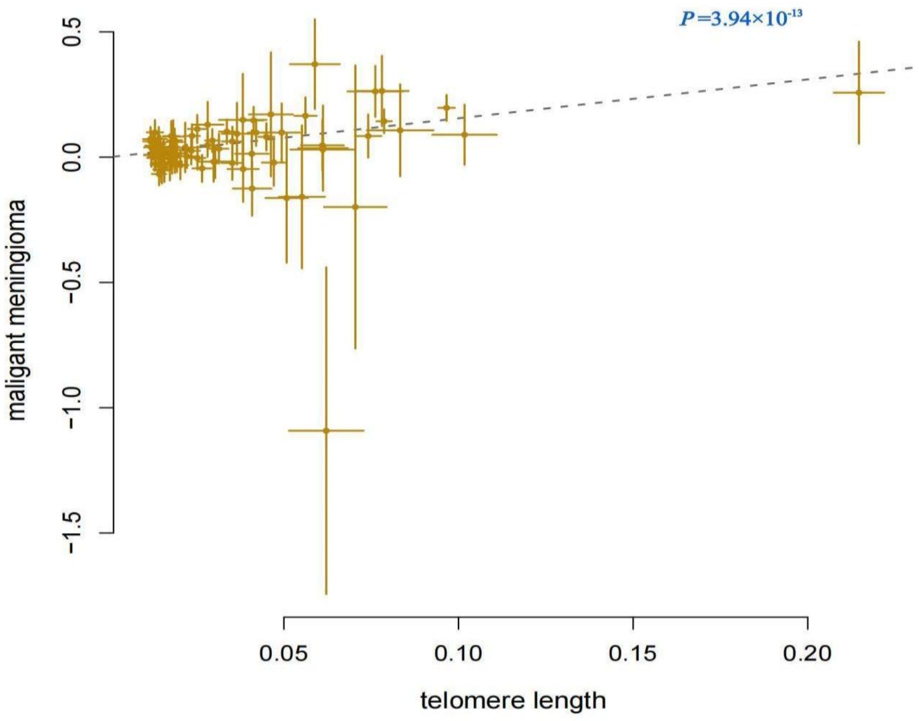

Supplementary figure 3_6_1 Illustration of forward Generalized summary data-based Mendelian randomization between LTL-472174 and malignant meningiomas

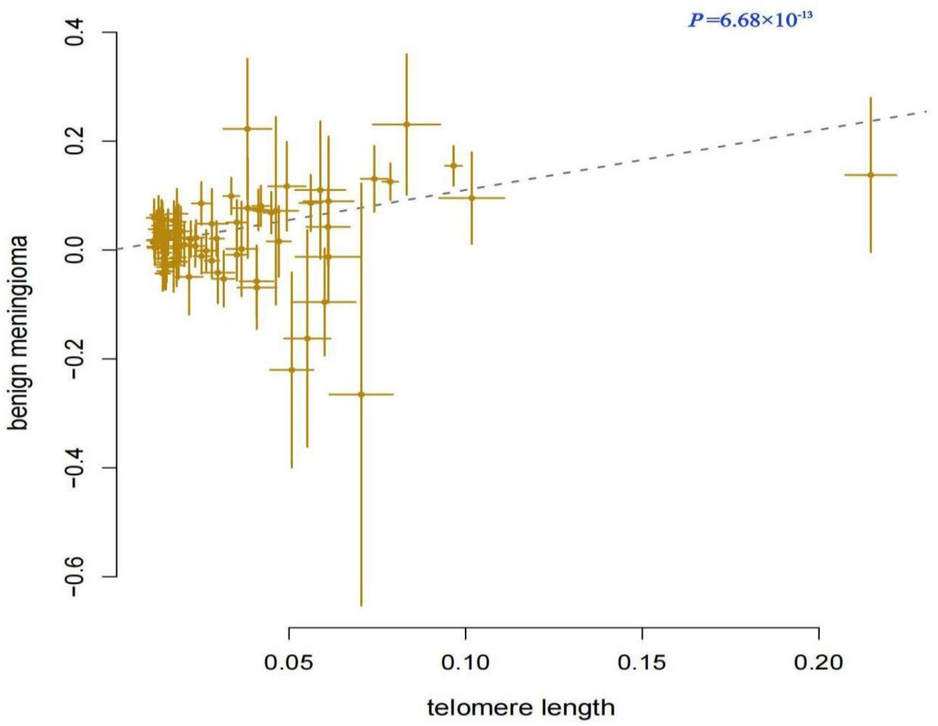

Supplementary figure 3_6_2 Illustration of forward Generalized summary data-based Mendelian randomization between LTL-472174 and benign meningiomas

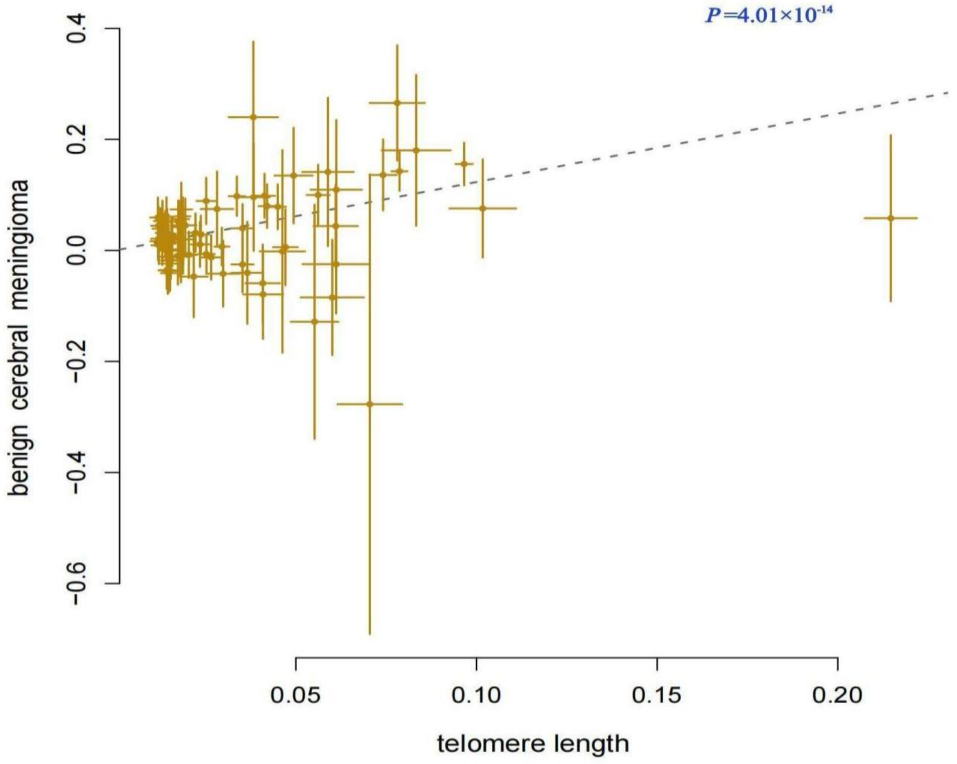

Supplementary figure 3_6_3 Illustration of forward Generalized summary data-based Mendelian randomization between LTL-472174 and benign cerebral meningiomas

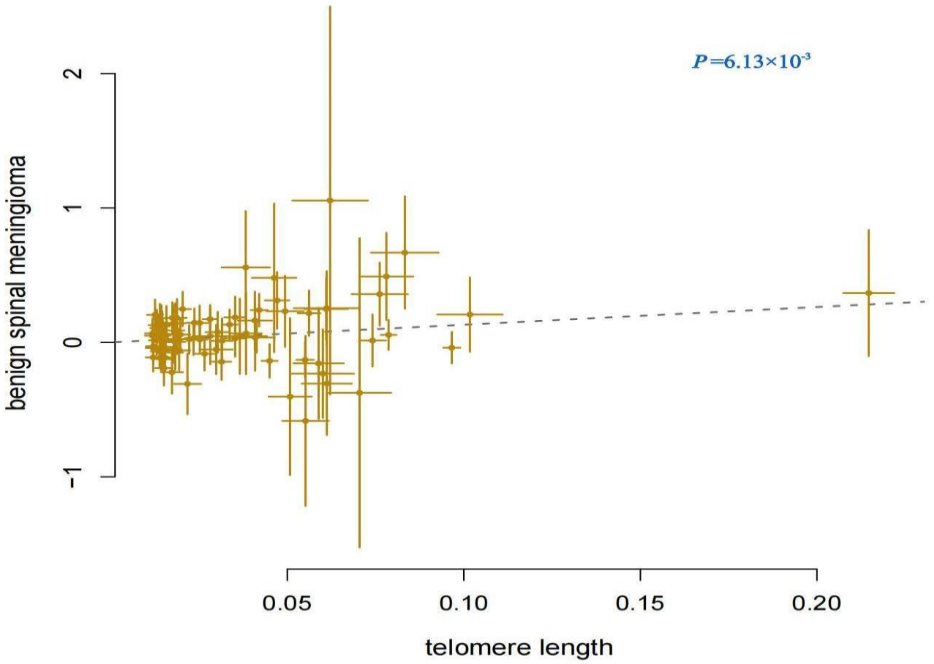

Supplementary figure 3_6_4 Illustration of forward Generalized summary data-based Mendelian randomization between LTL-472174 and benign spinal meningiomas

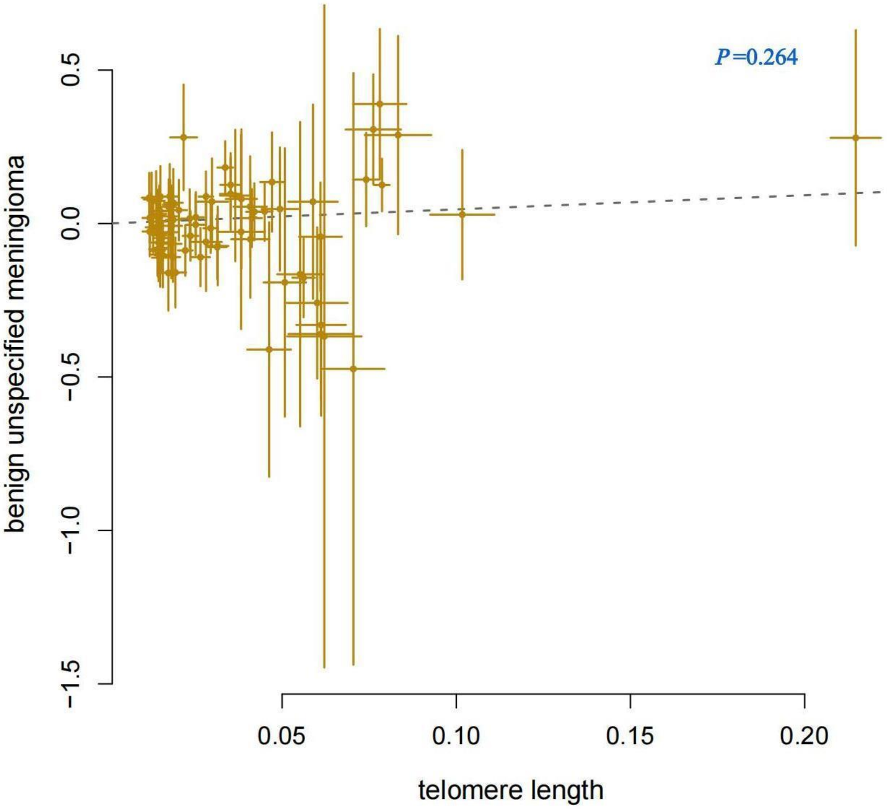

Supplementary figure 3_6_5 Illustration of forward Generalized summary data-based Mendelian randomization between LTL-472174 and benign unspecified meningiomas

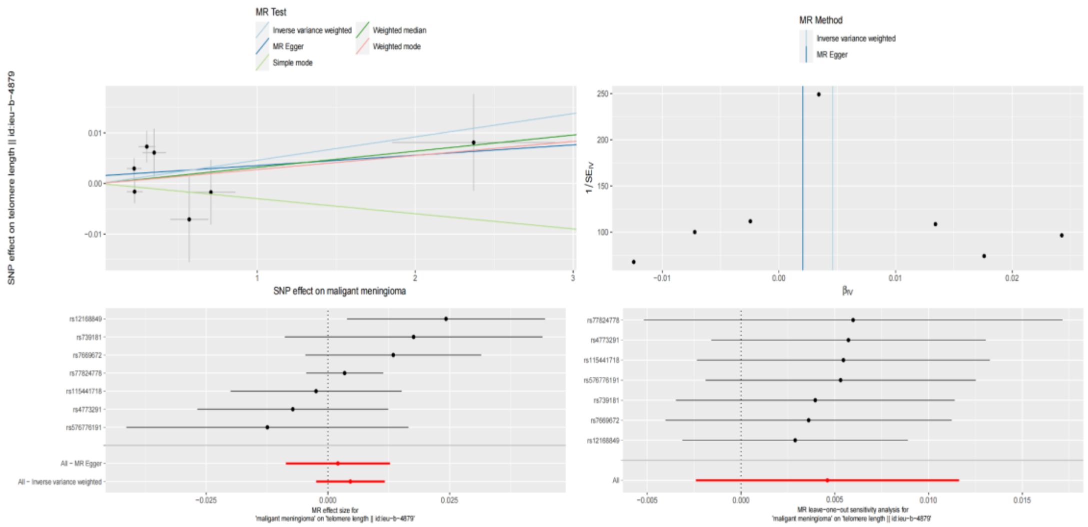

Supplementary figure 4_1 Illustrations of reverse Mendelian randomization and sensitivity analysis between LTL-472174 and malignant meningiomas. A: scatter plot; B: funnel plot; C: forest plot; D: leave-one-out analysis.

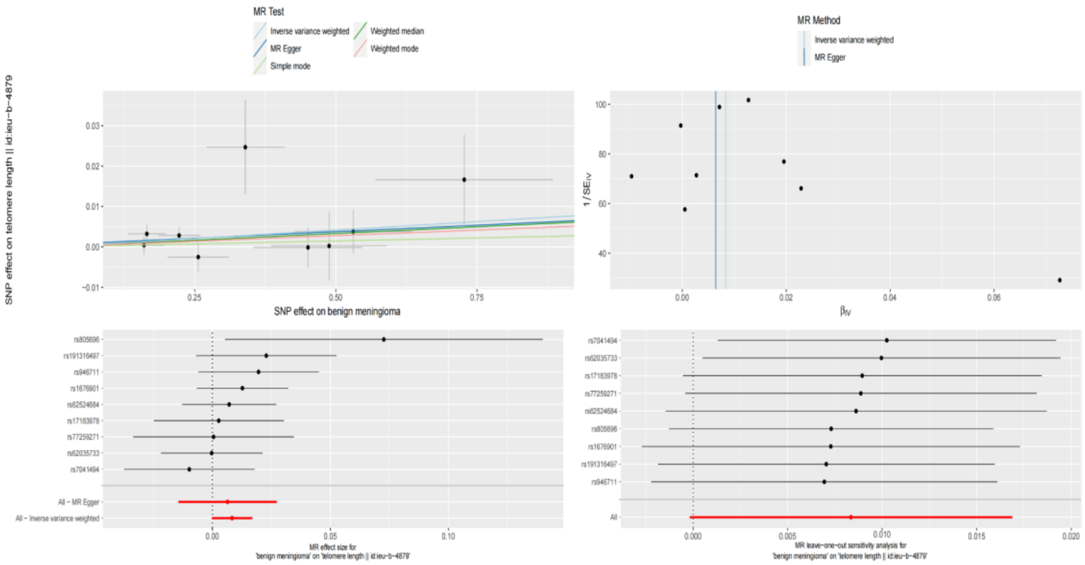

Supplementary figure 4_2 Illustrations of reverse Mendelian randomization and sensitivity analysis between LTL-472174 and benign meningiomas. A: scatter plot; B: funnel plot; C: forest plot; D: leave-one-out analysis.

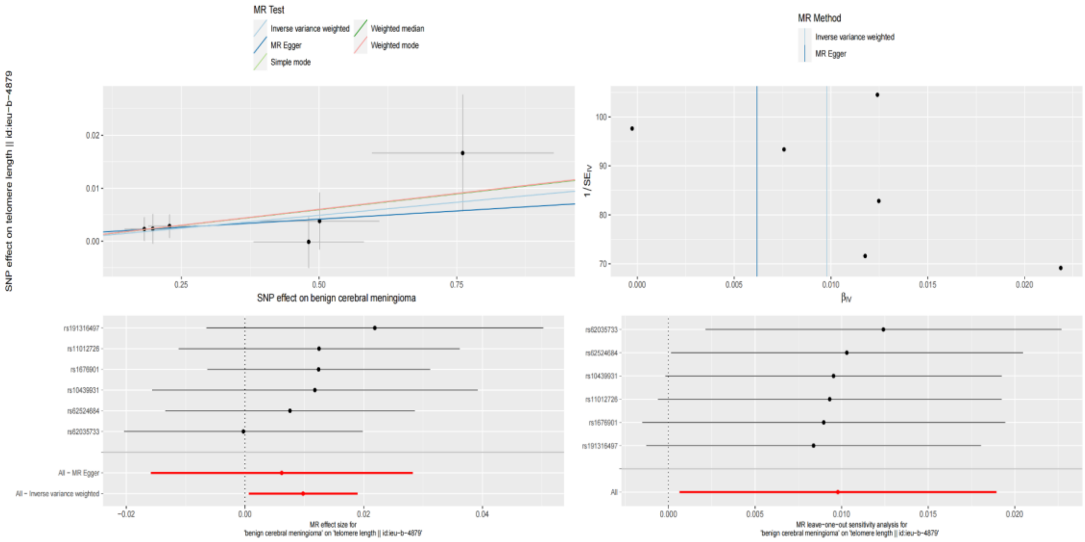

Supplementary figure 4_3 Illustrations of reverse Mendelian randomization and sensitivity analysis between LTL-472174 and benign cerebral meningiomas. A: scatter plot; B: funnel plot; C: forest plot; D: leave-one-out analysis.

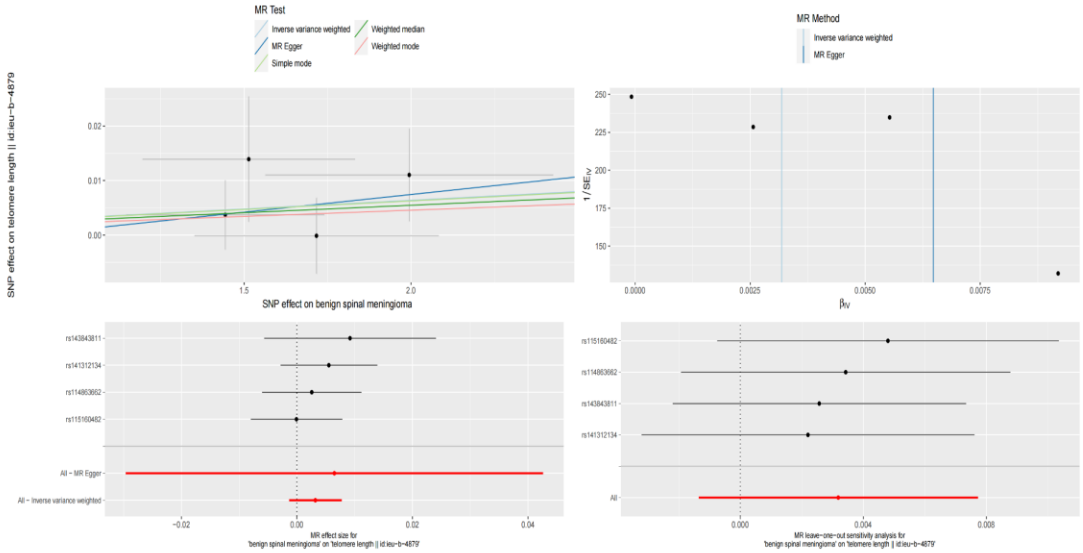

Supplementary figure 4_4 Illustrations of reverse Mendelian randomization and sensitivity analysis between LTL-472174 and benign spinal meningiomas. A: scatter plot; B: funnel plot; C: forest plot; D: leave-one-out analysis

.

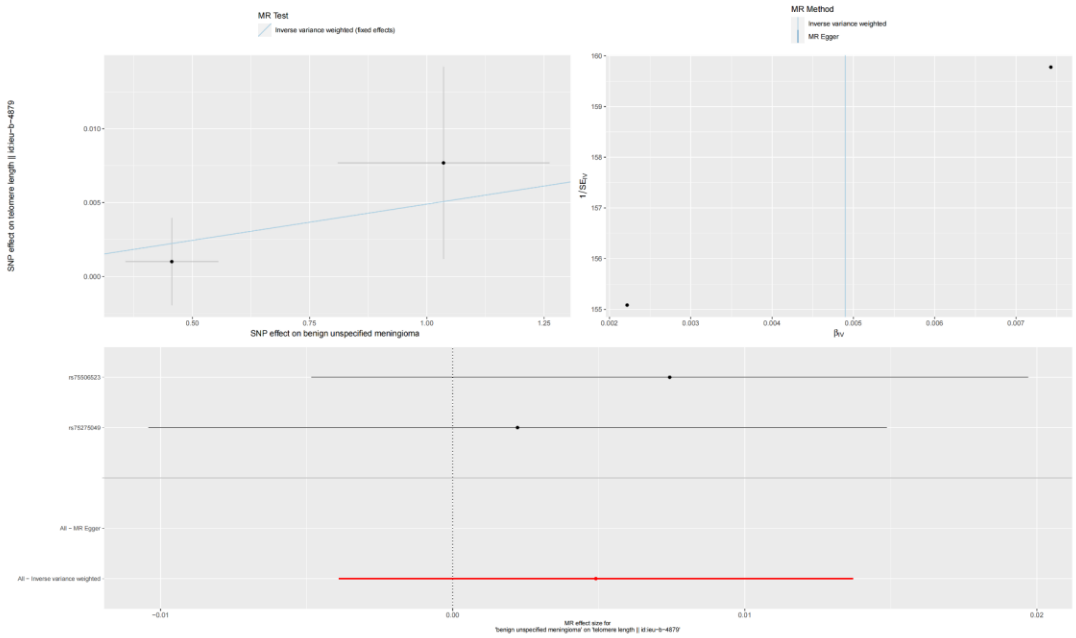

Supplementary figure 4_5 Illustrations of reverse Mendelian randomization and sensitivity analysis between LTL-472174 and benign unspecified meningiomas. A: scatter plot; B: funnel plot; C: forest plot.
